# Exploration of Target Spaces in the Human Genome for Protein and Peptide Drugs

**DOI:** 10.1016/j.gpb.2021.10.007

**Published:** 2022-03-23

**Authors:** Zhongyang Liu, Honglei Li, Zhaoyu Jin, Yang Li, Feifei Guo, Yangzhige He, Xinyue Liu, Yaning Qi, Liying Yuan, Fuchu He, Dong Li

**Affiliations:** 1 State Key Laboratory of Proteomics, Beijing Proteome Research Center, National Center for Protein Sciences (Beijing), Beijing Institute of Lifeomics, Beijing 102206, China; 2 School of Basic Medical Sciences, Anhui Medical University, Hefei 230032, China; 3 College of Chemistry and Environmental Science, Hebei University, Baoding 071002, China; 4 Suzhou Geneworks Technology Co., Ltd., Suzhou 215028, China; 5 Institute of Chinese Materia Medica, China Academy of Chinese Medical Sciences, Beijing 100700, China; 6 Department of Medical Research Center, Peking Union Medical College Hospital, Chinese Academy of Medical Science & Peking Union Medical College, Beijing 100730, China; 7 College of Life Sciences, Hebei University, Baoding 071002, China

**Keywords:** Protein drug, Peptide drug, Target analysis, Target prediction, Web server

## Abstract

After decades of development, protein and **peptide drugs** have now grown into a major drug class in the marketplace. Target identification and validation are crucial for the discovery of protein and peptide drugs, and bioinformatics prediction of targets based on the characteristics of known target proteins will help improve the efficiency and success rate of target selection. However, owing to the developmental history in the pharmaceutical industry, previous systematic exploration of the target spaces has mainly focused on traditional small-molecule drugs, while studies related to protein and peptide drugs are lacking. Here, we systematically explore the target spaces in the human genome specifically for protein and peptide drugs. Compared with other proteins, both successful protein and peptide drug targets have many special characteristics, and are also significantly different from those of small-molecule drugs in many aspects. Based on these features, we develop separate effective genome-wide **target prediction** models for protein and peptide drugs. Finally, a user-friendly **web server**, Predictor Of Protein and PeptIde drugs’ therapeutic Targets (POPPIT) (http://poppit.ncpsb.org.cn/), is established, which provides not only target prediction specifically for protein and peptide drugs but also abundant annotations for predicted targets.

## Introduction

After decades of development, protein and peptide drugs, whose emergence has revolutionized the pharmaceutical industry, have become a major drug class in the marketplace. Since the approval of the first recombinant protein drug in 1982 (Humulin, recombinant human insulin, Eli Lilly), the number of approved protein and peptide biopharmaceuticals has increased rapidly, from less than 10 in the 1980s to an approval rate of 10–12 per year (from 1995 to 2014), based on United States and European Union markets [1]. Thus far, more than 200 therapeutic proteins and peptides have been approved by the US Food and Drug Administration (FDA) [2]. A survey from 2004 to 2014 indicated that protein and peptide biopharmaceuticals represented about 25% of genuinely new drug approvals (excluding biosimilars, me-too products, and products previously approved elsewhere) in the United States [1]. Interestingly, in 2016 the FDA approved the lowest number of small-molecule drugs in nearly 45 years, resulting in the proportion of protein and peptide new molecule entity (NME) approvals reaching 40%, which is the highest to date [3]. Although in 2017 and 2018 the total number of new drug approvals was restored, the proportion of protein and peptide new drugs still remained at a high level (37% in 2017 and 30% in 2018) [4], [5]. Meanwhile, the market value of protein and peptide drugs is rising steadily. In 2018, of the top 10 best-selling drugs, 7 were therapeutic proteins or peptides (https://www.genengnews.com/a-lists/top-15-best-selling-drugs-of-2018/). Outlook by EvaluatePharma forecasts that the market share of biologics (the vast majority of which are protein and peptide drugs) was expected to increase from 25% in 2016 to 30% in 2022, and in 2022, 52% of the top 100 product sales would come from biologics [6].

Protein and peptide drugs currently include hormones, growth factors, blood factors, thrombolytics and anticoagulants, interferons and interleukins, and monoclonal antibodies, and have covered a wide range of therapeutic areas including cancer, various inflammation-related conditions, metabolic disorders, autoimmune diseases, and cardiovascular diseases [1], [7]. The success of protein and peptide-based therapeutics over the past several decades, in addition to associated developments in biotechnology, stems from their advantages over other drugs. Compared with traditional small-molecule drugs, protein and peptide drugs offer excellent target specificity, higher potency of action, fewer side-effects, and lower toxicity [8], [9], [10], and according to a survey from 2006 to 2015 [11], biologics have almost twice the success rate in clinical development as small-molecule NMEs. In particular, owing to their larger interaction contact surfaces, proteins and peptides are more suitable for targeting protein–protein interactions (PPIs) (which as potential drug targets have attracted much attention in recent years [12]) than small molecules.

Target identification is crucial for protein and peptide drug discovery, and bioinformatics prediction of candidate targets (*i.e.*, prediction of whether a protein is suitable as a drug target) based on the characteristics of known protein/peptide drug targets would be helpful for this process. A previous analysis showed that ∼ 70% of first-in-class drugs (*i.e.*, those that modulate an — until then — unprecedented target or biological pathway) were identified through a target-based drug discovery strategy [13]. In this strategy, target identification is the first step. Only after the target has been identified and validated can drug developers start to screen/design candidate drugs. Many experimental drug failures are attributed to inappropriate target selection, consuming a large number of resources [14], [15]. It is vital to provide as much evidence as possible to support a target choice before investing further resources into that target [16]. In addition to disease relevance, successful drug targets generally have some common features which are different from other nontarget proteins. It is of great important to systematically explore the features of successful drug targets and further to computationally predict candidate targets based on these features. This will not only contribute to the understanding of the cellular roles of targets and the molecular mechanism of action (MoA) of drugs from an overall perspective, but also greatly increase the efficiency and success rate of target selection.

Owing to the developmental direction of the pharmaceutical industry, up to now the systematic analyses of known targets and the prediction of candidate targets have mainly focused on traditional small-molecule drugs, while studies related to protein and peptide drugs are still lacking. In previous studies, the properties of target proteins such as protein families, biochemical functions, subcellular locations, network topology, and sequences have been explored [16], [17], [18], [19]. Furthermore, some target prediction methods have been developed [16], [18], [19], [20], [21]. For example, early common prediction strategies included those based on domain family affiliation [21], and those designed by searching binding pockets on the protein surface based on 3D structures to identify proteins that may bind to small-molecule drugs [22]. Recently, some studies have used machine learning methods to establish target prediction algorithms on the basis of summarized target features of protein sequences, physicochemical properties, network topology, *etc.* [16], [20], [23], [24]. In addition, several online tools for target prediction have also been established such as DrugMiner [20] and D3TPredictor [25]. However, small-molecule drug targets dominated all these studies, and the systematic exploration of the target space specifically for protein/peptide drugs is still lacking.

It is necessary to systematically explore the target spaces in the human genome specifically for protein and peptide drugs because of the potential differences that may exist between the targets of different types of drugs. Realizing this potential difference, Sanseau et al*.* presented a protocol for distinguishing potential targets of protein drugs from those of small-molecule drugs in genes derived from a genome-wide association study (GWAS), using the signal peptide and transmembrane domain features for the former and the domain family distribution features for the latter [26]. Similarly, Jeon et al*.* roughly distinguished putative cancer targets for protein, peptide, and small-molecule drugs individually using extracellular domain inclusion, peptide-binding domain inclusion, and the existence of corresponding small-molecule inhibitors [27]. Zhu et al*.* found that the targets of biologics have higher degrees and smaller cluster coefficients than those of small-molecule drugs [28]. However, in these studies, the authors did not perform any systematic analyses/comparisons of targets of protein, peptide, and small-molecule drugs and did not construct any drug target prediction models.

**Figure 1:**
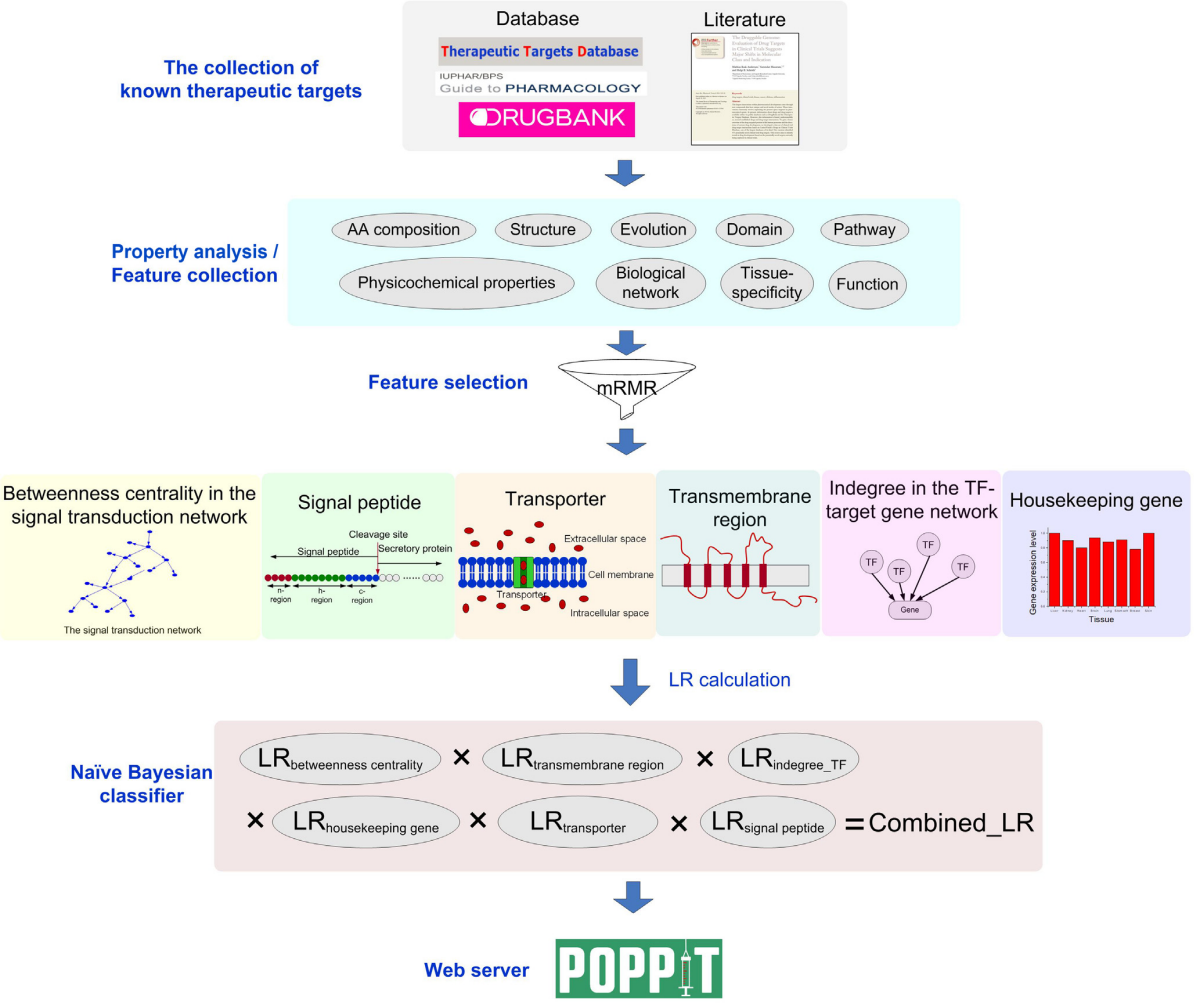
**Workflow for exploring the target spaces for protein drugs** Firstly the dataset of therapeutic targets of approved protein drugs was established by integrating data from the related databases and literature. Then by comparison with other nontarget proteins from multiple aspects, the features of protein drug targets were revealed. Next, we used mRMR feature selection method to rank these features and then used a naïve Bayesian classifier to integrate six representative features to establish the target prediction model for protein drugs. Finally the model was developed into a web server. LR, likelihood ratio; mRMR, minimum redundancy maximum relevance; AA, amino acid; TF, transcription factor.

Considering these issues, in this paper we have systematically explored target spaces in the human genome specifically for protein and peptide drugs (Figure 1). First, we comprehensively collected the therapeutic targets of approved protein and peptide drugs. Then, their characteristics were separately systematically analyzed considering multiple aspects and compared with those of conventional small-molecule drugs. Next, after feature selection, we used a naïve Bayesian classifier to integrate several representative features to build the target prediction models separately for protein and peptide drugs. Finally, to facilitate the usage of the prediction models, a user-friendly web server, the Predictor Of Protein and PeptIde drugs’ therapeutic Targets (POPPIT), was established, supporting online target prediction for protein and peptide drugs and providing various related annotations for predicted targets.

## Method

### Therapeutic targets

Here, we only collected therapeutic targets of approved drugs. Therapeutic targets are defined as those proteins (or other biomolecules) that a drug targets to exert its therapeutic effects, excluding side-effect targets and other binding proteins without pharmacological efficacy [29], [30]. We collected therapeutic targets and their corresponding approved drugs (excluding withdrawn drugs) from the Therapeutic Target Database (TTD, version: 5.1.02) [31], GtoPdb (downloaded on March 19, 2017) [32], and DrugBank (downloaded on July 26, 2015) [33] databases, as well as from the study by Rask-Andersen and colleagues [29]. For GtoPdb, we only considered its strictly defined “primary” targets, and for DrugBank, only drug targets with known pharmacological action were considered. All targets of the “group” type, such as protein complexes, were excluded. Drug types (protein, peptide, or small-molecule drugs) were distinguished based on the related annotations provided by corresponding data sources or by manual curation, where peptide drugs were separated from proteins on the basis of size and were arbitrarily defined as molecules containing fewer than 50 amino acids (AAs) [8]. In total, we obtained 132, 55, and 634 human therapeutic target proteins uniformly represented by Swiss-Prot accession number (SP AC) for protein, peptide, and small-molecule drugs, respectively (Tables S1–S3). Finally, to avoid potential bias, any homologous redundancy (sequence identity > 40% [34]) was removed, leaving 99, 39, and 359 target proteins for protein, peptide, and small-molecule drugs, respectively, which were used for the property investigation and the prediction model construction of protein and peptide drug targets.

### Gold standard datasets

To establish target prediction models, first, the gold standard positive (GSP) and gold standard negative (GSN) datasets were constructed for the protein and peptide drugs, respectively. The GSP set for protein drugs was the non-redundant target set of protein drugs established above. For the GSN set for protein drugs, considering the difficulty in developing an experimentally negative dataset, we adopted the following construction scheme. First, we removed as many known protein drug targets as possible from the whole human genome. To ensure that the GSN set was as “pure” as possible, here, the removed known targets not only included the targets collected above but also other possible targets. These other possible targets included targets of trial protein drugs from the study by Rask-Andersen et al. [29], non-“primary” targets of approved protein drugs in GtoPdb (downloaded on March 19, 2017), and all targets (also including those without known pharmacological action) of all protein drugs (also including experimental drugs) in DrugBank (downloaded on July 26, 2015), as well as those of the “group” type such as targets of protein complex type. Then, from the remaining proteins, we randomly selected 100 non-redundant ones to construct the GSN set. Given the randomness during the GSN set construction, to avoid potential bias we repeated the construction process of the GSN set 100 times and finally reported the mean ± standard deviation (SD) of the results. The construction of the GSP and GSN sets for peptide drugs used a similar protocol to that for the protein drugs (the size of the GSN set was 100).

### Independent test datasets

To assess the performance of the prediction models, in addition to cross-validation, three independent test sets were also established.

The first independent positive test set was composed of the newly added therapeutic target set of the approved protein/peptide drugs from the latest version of DrugBank (released on July 3, 2018). After removing the overlap with the GSP and GSN sets, 45/29 targets remained to constitute this independent positive test set for protein/peptide drugs.

The second independent positive test set was composed of 141/60 targets of clinical trial protein/peptide drugs from the study by Rask-Andersen and colleagues [29] (after removing the overlap with the GSP and GSN sets).

In the third independent positive test set, we divided the GSP set of the protein/peptide drug targets into two parts. One part was composed of drug targets approved before 2010 (as well as those without approval date information). This part together with the GSN set built above was used to train the prediction model. The other part involved new therapeutic targets introduced by drugs approved in or after 2010, and was used as the independent positive test set. The approval date information of drugs was obtained from related annotations of corresponding data sources or identified by Drugs@FDA (https://www.accessdata.fda.gov/scripts/cder/daf/).

The independent negative test set for each of these three groups of positive test sets was established in a similar way to the construction of the GSN set. Specifically, before randomly choosing a similar number of proteins to that of the corresponding independent positive test set, in addition to removing the known protein/peptide drug targets, the corresponding GSN set and the independent positive test set were also excluded. Considering the randomness of the construction of the independent negative test set, we also repeated the construction process 100 times. Finally, the assessment results of the prediction models on independent test sets were calculated as the mean ± SD of 10,000 results (*i.e.*, the results of 100 prediction models constructed based on 100 gold standard datasets against 100 independent test sets).

### Data sources and methods related to property analyses of therapeutic targets

AAs were classified into 9 groups: tiny (A+C+G+S+T), small (A+B+C+D+G+N+P+S+T+V), aliphatic (A+I+L+V), aromatic (F+H+W+Y), nonpolar (A+C+F+G+I+L+M+P+V+W+Y), polar (D+E+H+K+N+Q+R+S+T+Z), charged (B+D+E+H+K+R+Z), basic (H+K+R), and acidic (B+D+E+Z). Here, “B” is the abbreviation of “Aspartate or Asparagine” and “Z” is the abbreviation of “Glutamate or Glutamine” [35]. We used the pepstats program in EMBOSS (version 6.5.0) to compute the percentage of each class of AAs and the charge of a protein sequence [35]. The grand average of hydropathy (GRAVY) value of a protein was computed as the sum of hydropathy values [36] of its AAs, divided by its AA sequence length. The GRAVY value and theoretical isoelectric point (pI) were calculated by the ProtParam program downloaded from the Comprehensive Perl Archive Network (CPAN) (https://www.perl.org/cpan.html). The AA sequences of human proteins were obtained from Swiss-Prot (downloaded on January 11, 2015) [37].

Pfam domain [38] assignments of human proteins were parsed from Swiss-Prot (downloaded on March 23, 2016). Intrinsically disordered proteins are those that lack fixed or ordered 3D structures. The disorder score of a protein was computed as the ratio of the length of the disordered regions to its total length [39]. FoldIndex was used to predict the intrinsic disorder of a protein (using default parameters) [40] and to reduce the false positive rate, only those disordered regions with length ≥ 30 were considered [39]. A PEST region is a peptide sequence enriched in proline (P), glutamic acid (E), serine (S), and threonine (T) and is invariably found in proteins with a short half-life and thus hypothesized to serve as a proteolytic signal [41]. Here, we adopted the EMBOSS epestfind program (using default parameters) [35] to count the number of PEST motifs in a protein, and only “potential” PEST motifs were included. A signal peptide is a short peptide present at the N-terminus of a protein, which is targeted to the endoplasmic reticulum and eventually destined to be either secreted, extracellular, or periplasmic [37]. Signal peptides and transmembrane regions of human proteins were both parsed from related annotations of Swiss-Prot (downloaded on February 11, 2016).

The tissue specificity score (TSPS) was adopted to measure the degree of tissue specific expression of a gene [42]. The TSPS of a gene was computed as:

(1) $$\text{TSPS =}\;\sum_{i}f_{i}\log_{2}\left(f_{i}/p\right)$$

where *f_i_* is the gene expression level of the gene in tissue *i* divided by the sum of expression levels of the gene across all tissues, and *p* = 1/*n* (*n* is the number of tissues). The larger the TSPS value is, the more tissue-specific the expression of the gene is. Here we used RNA-sequencing data from 32 tissues provided by Uhlén et al*.* [43] to compute the TSPS.

The data on evolutionary rates and original ages of human proteins were obtained from our previous work [44]. Human proteins were grouped into 16 age classes, from the oldest “Cellular organisms” class of age 16 (composed of proteins that originated from the common ancestor of the three domains of the tree of life: Eukaryota, Bacteria, and Archaea) to the youngest “*Homo sapiens*” class of age 1 (whose proteins are only found in humans). We used *C_ratio_* to check the gene polymorphism, which was computed as:

(2) $$C_{\mathrm{ratio}}=\frac{N_{\mathrm{ns}}+\varepsilon }{N_{s}+\varepsilon }$$

where *N_ns_* and *N_s_* are the number of nonsynonymous and synonymous single nucleotide polymorphisms (SNPs) in the gene, respectively, and *ε* was set to 0.01 as per Yao et al*.* [23] to eliminate statistical aberrations induced by the relatively small sample size. Coding-region SNP data were obtained from the dbSNP database (downloaded on October 18, 2016) [45], and only SNPs with a global minor allele frequency (GMAF) no smaller than 1% were considered.

The list of 1639 human transcription factors (TFs) was from the study by Lambert and colleagues [46]. Housekeeping genes are those detected in all tissues, which were obtained based on RNA-sequencing data in 32 tissues from the study by Uhlén and colleagues [43], involving 8874 genes. Lists of signaling molecules and self-interacting proteins were from the signal transduction network and the integrated PPI network, respectively, described in the next paragraph. The human genes coding enzymes were parsed from Swiss-Prot (downloaded on February 11, 2016), G protein-coupled receptors (GPCRs) and kinases from UniProt (http://www.uniprot.org/docs/7tmrlist and http://www.uniprot.org/docs/pkinfam) (released on October 25, 2017) [37], both ion channels and nuclear hormone receptors (NHR) from the HUGO Gene Nomenclature Committee (HGNC) database (downloaded in July, 2017) [47], and transporters from the Human Transporter Database (HTD) (version: January 1, 2014) [48].

Human gene–reaction associations were from Recon 2 (version: November 5, 2015) [49], and biological pathways were from KEGG (downloaded on July 13, 2016) [50]. The degree of a node in the network is equal to the number of its interaction partners. Betweenness centrality was computed as:

(3) $$B\left(n\right)=\frac{2\times \sum_{s\neq n\neq t}\frac{\sigma_{\mathrm{st}}\left(n\right)}{\sigma_{\mathrm{st}}}}{\left(N-1)(N-2\right)}$$

where *σ_st_* represents the number of the shortest paths between nodes *s* and *t*, *σ_st_*(*n*) is the number of those paths that go through node *n*, and *N* is the total number of nodes in the network. Degree reflects a node’s local importance, while betweenness centrality captures the degree to which the node influences the communication between other nodes in the network [51]. The human PPI network was obtained by integrating experimental PPIs of the “direct interaction” type from multiple databases (File S1). The human signal transduction network was provided by Cui et al. (version 6) [52], and the transcriptional regulation network was obtained from the study by Chouvardas and colleagues [53]. Here, the PPI and signal transduction networks were regarded as undirected networks, while the transcriptional regulation network was regarded as a directed network. In the directed transcriptional regulation network, the indegree (of a target gene) denotes the number of TFs regulating this target gene, while the outdegree (of a TF) the number of target genes regulated by this TF.

### Minimum redundancy maximum relevance feature selection

We used the minimum redundancy maximum relevance (mRMR) method for feature selection. mRMR ranks features based on both their relevance to the classification variable and the redundancy between each other [51], [54]. Both the relevance and redundancy were quantified by mutual information (MI), denoted by *I* (File S1).

Suppose that *S* and *T* are separately the already-selected and to-be-selected feature sets and |*S*| and |*T*| are the number of features in the corresponding sets. mRMR first moves the feature most relevant to the classification variable into *S* and then moves the remaining features from *T* into *S* one by one, requiring that each time the selected feature *f_j_* optimizes:

(4) $$\underset{}{\max_{f_{j}\in T}}\left(\frac{I(f_{j},c)}{\frac{1}{\left|S\right|}\sum_{f_{i}\in S}I(f_{j},f_{i})}\right)$$

where $I(f_{j},c)$ is the relevance of the candidate feature *f_j_* to the classification variable *c* and $\frac{1}{\left|S\right|}\sum_{f_{i}\in S}I(f_{j},f_{i})$ is its redundancy with features already in *S*.

The GSN set was repeatedly constructed 100 times as stated above, and thus, the mRMR feature ranking was implemented based on the mean MI of the 100 times. The mRMR program was obtained from http://home.penglab.com/proj/mRMR/.

### Naïve Bayes classifier

According to the Bayes rule, the posterior odds *O_post_* that a protein is a target can be computed as the product of its prior odds *O_piror_* and the likelihood ratio (LR), represented by Equations ((5), (6), (7)), where P(positive) and P(negative) are, respectively, the probabilities that a protein is and is not a drug target, and $P(positive|f_{1}...f_{n})$ and $P(negative|f_{1}...f_{n})$ are the corresponding probabilities after considering *n* biological evidence types (*i.e.*, features).

(5) $$O_{\mathrm{post}}=O_{\mathrm{prior}}\times \text{LR}\left(f_{1}...f_{n}\right)$$

(6) $$O_{\mathrm{piror}}=\frac{P(positive)}{P(negative)}$$

(7) $$O_{\mathrm{post}}=\frac{P(positive|f_{1}...f_{n})}{P(negative|f_{1}...f_{n})}$$

The LR of biological evidence *f* is referred to as the ratio of the probability of feature *f* being observed in the GSP set to that in the GSN set:

(8) $$\text{LR}\left(f\right)=\frac{P(f|positive)}{P(f|negative)}=\frac{TP_{f}/T}{FP_{f}/F}$$

where *T* and *F* are the number of true and false drug targets in the gold standard dataset, and *TP_f_* and *FP_f_* are the number of those satisfying evidence *f*. The LR can reflect the prediction ability of biological evidence *f*. When the LR is larger than 1, *O_post_* is larger than *O_piror_*. This means that the feature has some ability to discriminate true drug targets from false ones. When the integrated *n* features are independent, the naïve Bayes rule can be adopted, where the combined_LR can be calculated simply as the product of the LRs of individual features:

(9) $$\text{LR}\left(f_{1}...f_{n}\right)=\prod_{i=1}^{i=n}\text{LR}(f_{i})$$

The prior odds *O_piror_* is actually constant for different proteins, and thus *O_post_* is proportional to the LR. In practice, we directly used the LR as the prediction score to reflect the probability that a protein is a drug target [55], [56].

The naïve Bayes classifier was implemented by Perl scripts written in-house.

#### Performance assessment

We used the receiver operating characteristic (ROC) curve to measure the performance of the prediction model based on 10-fold cross-validation and the independent test sets (File S1). Here, the ROC curves were plotted using SPSS software [57].

#### Implementation of the POPPIT web server

The foundation of the POPPIT web server was a MongoDB database. Above this database, the analysis application was implemented in Perl and Python, and the web presentation application was written in JavaScript and CSS.

## Results

### The statistics of known therapeutic targets

To systematically explore the target spaces of protein and peptide drugs, we first comprehensively collected the therapeutic targets of approved drugs. In total, we obtained 132, 55, and 634 target proteins for approved protein, peptide, and small-molecule drugs, respectively (see Method) (Tables S1–S3).

**Figure 2:**
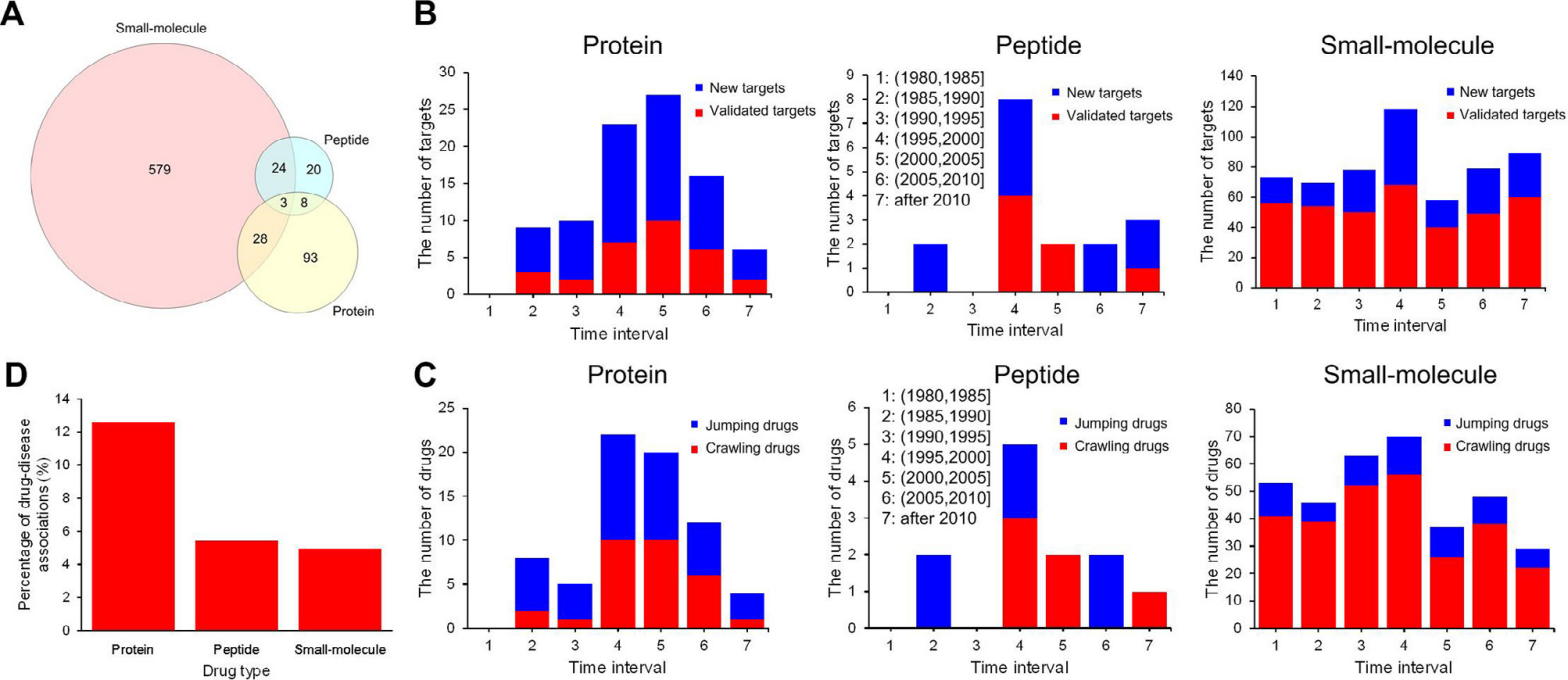
**Statistics on the therapeutic targets of approved protein, peptide, and small-molecule drugs** **A.** A Venn graph of the therapeutic targets of the three types of drugs. The number on each part on the graph is the number of targets. **B.** The composition of therapeutic targets of new drugs approved every five years. “Validated targets” refer to those that have been used as targets of previously approved drugs, while “new targets” have not. Each bin represents five years, and the year scope of each bin is described on the graph. **C.** The composition of drugs newly approved every five years. We used the definition by Yildirim et al*.*[58], where “jumping drugs” are those with totally new target sets, and “crawling drugs” have at least one “validated target”. The analyses of (B) and (C) only included drugs with at least one therapeutic target, and these results were obtained based on therapeutic targets of approved drugs from the study by Rask-Andersen et al. [29] (see Method). We obtained consistent conclusions based on the data from DrugBank (downloaded on July 26, 2015, see Method) (Figure S1). **D.** The percent of known drug–disease associations where at least one therapeutic target of the drug is simultaneously the known related gene of the disease. Here we only considered those known drug–disease associations involving drugs with at least one target and diseases with at least one known related gene (File S1).

First, we noticed that although there was overlap, most targets were specific to a certain drug type (protein, peptide, or small-molecule drugs) (Figure 2A), suggesting potential differences between the target spaces of different types of drugs.

Next, we added approval date labels for the drugs, and from the perspective of targets, we compared the innovation power of the three different types of drugs. We found that for protein drugs, on average, over half (68.06%) of the targets of drugs newly approved every five years were “new targets”, while this average proportion (32.29%) was much smaller for small-molecule drugs (rank sum test, *P* = 0.0010) (Figure 2B). “New targets” are referred to as those that have not been used as targets before for any approved drug [58]. The analyses of the new drug composition also led to a consistent tendency, that is, the average percent of “jumping drugs” was much higher among protein drugs than that among small-molecule drugs (64.09% > 21.43%, rank sum test, *P* = 0.0010) (Figure 2C), where “jumping drugs” are those new drugs whose targets are all “new targets”; otherwise, they are called “crawling drugs” [58]. For peptide drugs, although the average proportion of “new targets” (Figure 2B) or “jumping drugs” (Figure 2C) was larger than that of small-molecule drugs, we did not observe a statistically significant difference. These results indicate that new protein drugs tend to bind to “new targets” rather than to validated targets and have stronger target innovation power than traditional small-molecule drugs.

Finally, we analyzed and compared the drug–disease relationship for different types of drugs. This analysis was performed by simply counting the proportion of known drug–disease associations where at least one of the drug’s therapeutic targets is also the known related gene of the corresponding disease (File S1). We found that this proportion for protein drugs was significantly larger than that for small-molecule drugs (12.60% > 4.96%, Fisher’s exact test, *P* = 2.20E–16) (Figure 2D). This indicates that compared with small-molecule drugs, protein drugs tend to directly target disease-causing proteins. This confirms to some degree the previous hypothesis that compared with small-molecule drugs, protein drugs tend to cure diseases rather than only treat their symptoms [10]. However, we did not observe a significant difference between peptide and small-molecule drugs (Figure 2D).

### The targets of protein and peptide drugs have distinctive properties

In this section, we intend to answer two questions. One is “what characteristics do protein and peptide drug targets have with respect to other proteins, respectively”. The other is “are they different from conventional small-molecule drug targets, and if so, how”.

For the first question, in terms of sequence and physicochemical property, we found that compared with other, nontarget proteins, successful protein drug targets tend to contain significantly higher proportions of tiny, small, and aromatic AAs, lower proportions of basic and charged AAs, and they are significantly more hydrophobic (measured by GRAVY), and their charges tend to be negative and therefore have a significantly lower theoretical pI (Table 1).

**Table 1:** **Quantitative differences between protein drug targets and other proteins**

**Property**	**Mean value (mean rank)**		***P* value** **(rank sum test,** **one-sided)**	**Adjusted** ***P* value**
	**Protein drug** **targets**	**Other** **proteins**		
Tiny (%)	29.9664 (5066)	29.2258 (4536)	**2.26E–02**	**3.40E–02**
Small (%)	51.6968 (5623)	49.4238 (4530)	**1.84E–05**	**3.82E–05**
Aliphatic (%)	27.5851 (4132)	28.1409 (4546)	5.90E–02	7.56E–02
Aromatic (%)	10.8935 (5107)	10.4023 (4535)	**1.55E–02**	**2.61E–02**
Non-polar (%)	54.4097 (4945)	53.4460 (4537)	6.16E–02	7.56E–02
Polar (%)	45.5903 (4138)	46.5540 (4546)	6.16E–02	7.56E–02
Charged (%)	22.9411 (3212)	25.4849 (4556)	**1.96E–07**	**5.88E–07**
Basic (%)	12.0871 (2823)	14.1462 (4560)	**2.74E–11**	**1.23E–10**
Acidic (%)	10.8539 (4178)	11.3386 (4546)	8.25E–02	9.28E–02
GRAVY	–0.2596 (5066)	–0.3324 (4535)	**2.26E–02**	**3.40E–02**
Theoretical pI	6.5817 (3398)	7.3504 (4554)	**6.48E–06**	**1.46E–05**
Charge	–2.4444 (3336)	4.0814 (4555)	**2.10E–06**	**5.15E–06**
Domain number	3.3333 (6422)	1.4393 (4521)	**1.16E–15**	**6.26E–15**
Disorder score	0.1799 (4135)	0.2535 (4546)	5.80E–02	7.56E–02
PEST motif number	0.6364 (4729)	0.6232 (4539)	1.94E–01	2.09E–01
TSPS	1.3393 (5742)	0.9524 (4158)	**5.00E–11**	**1.93E–10**
Age	8.5500 (2107)	10.9609 (3434)	**8.00E–10**	**2.70E–09**
Evolutionary rate	5.5031 (3518)	4.4871 (3204)	6.94E–02	8.14E–02
*C_ratio_*	38.5784 (3680)	34.3152 (3644)	4.36E–01	4.36E–01
Reaction number	0.0404 (4265)	0.4614 (4545)	**1.28E–02**	**2.31E–02**
Pathway number	6.1327 (7362)	0.9661 (4243)	**2.20E–16**	**1.49E–15**
Degree_ PPI	9.9565 (3416)	8.7294 (2823)	**2.51E–04**	**4.84E–04**
Betweenness centrality_ PPI	0.0002 (3607)	0.0001 (2820)	**1.95E–06**	**5.15E–06**
Degree_signal	40.8778 (1807)	15.6977 (1175)	**2.20E–16**	**1.49E–15**
Betweenness centrality_signal	0.0012 (1852)	0.0002 (1173)	**2.20E–16**	**1.49E–15**
Indegree_TF	7.4815 (1186)	2.9613 (784)	**2.20E–16**	**1.49E–15**
Outdegree_TF	4.0000 (148)	10.2581 (128)	2.16E–01	2.24E–01

*Note*: Tiny (%), small (%), aliphatic (%), aromatic (%), non-polar (%), polar (%), charged (%), basic (%), and acidic (%) refer to the proportions of tiny, small, aliphatic, aromatic, non-polar, polar, charged, basic, and acidic AAs in the whole protein sequence, respectively. Domain number is counted for each protein including domain repeats. “Degree_PPI” and “Betweenness centrality_PPI” are degree and betweenness centrality of a protein in the PPI network, respectively. “Degree_signal” and “Betweenness centrality_signal” are separately degree and betweenness centrality in the signal transduction network. “Indegree_TF” and “Outdegree_TF” are indegree and outdegree of a gene in the TF–target gene network, where the indegree of a (target) gene is the number of TFs regulating the gene and outdegree of a gene (*i.e.*, TF) is the number of target genes regulated by the TF. *P* values and adjusted *P* values smaller than 0.05 are represented in bold. Adjusted *P* values are computed by Benjamini-Hochberg multiple testing correction method. GRAVY, grand average of hydropathy; pI, isoelectric point; PPI, protein–protein interaction; TSPS, tissue specificity score; TF, transcription factor; AA, amino acid; PEST, a peptide sequence enriched in proline (P), glutamic acid (E), serine (S), and threonine (T).

In terms of structure, compared with other proteins, protein drug targets contain significantly more domains (Table 1), indicating that they tend to have more complex structures and functions. Furthermore we observed that compared with other proteins, significantly more protein drug targets contain transmembrane regions and signal peptides (Table 2), which is consistent with previous knowledge [26].

**Table 2:** **Qualitative differences between protein drug targets and other proteins**

**Property**	**The fraction of proteins belonging** **to a certain protein class (%)**		***P* value** **(Fisher’s exact test,** **one-sided)**	**Adjusted** ***P* value**
	**Protein drug** **targets**	**Other** **proteins**		
Protein with signal peptide	84.85	15.24	**3.39E–52**	**4.07E–51**
Protein with transmembrane region	66.67	24.60	**2.25E–18**	**9.01E–18**
Signaling molecule	90.91	25.70	**3.29E–42**	**1.97E–41**
Transcription factor	0.00	2.97	5.13E–02	7.69E–02
Housekeeping gene	24.24	50.52	**9.39E–08**	**2.82E–07**
Self-interacting protein	22.22	8.59	**3.35E–05**	**8.04E–05**
Enzyme	15.15	20.92	9.68E–02	1.29E–01
GPCR	5.05	1.87	**4.04E–02**	6.93E–02
Ion channel	0.00	1.04	3.59E–01	3.92E–01
NHR	0.00	0.13	8.77E–01	8.77E–01
Kinase	3.03	1.44	1.74E–01	2.09E–01
Transporter	0.00	5.48	**3.91E–03**	**7.82E–03**

*Note*: *P* values and adjusted *P* values smaller than 0.05 are represented in bold. Adjusted *P* values are computed by Benjamini-Hochberg multiple testing correction method. GPCR, G protein-coupled receptor; NHR, nuclear hormone receptor.

In terms of gene expression and evolutionary aspects, compared with nontarget proteins, protein drug targets tend to be tissue-specific (measured by TSPS) (Table 1), which may lead to less potential side effects. Furthermore protein drug targets originated significantly later. Although they evolved more quickly, the difference did not reach statistical significance (Table 1). There was also no apparent difference between protein drug targets and other proteins in terms of gene nonsynonymous polymorphism (measured by *C_ratio_*) at the human population level (Table 1).

Furthermore in terms of functional aspects, 91% of the collected protein drug targets participate in signal transduction, several times higher than the proportion of other proteins (Table 2). Housekeeping genes are also significantly lacking (Table 2), consistent with the high tissue specificity of protein drug targets. Protein drug targets are significantly enriched with self-interacting proteins (Table 2), in line with our previous observation [51]. In terms of biochemical function, protein drug targets have special characteristics. Previous studies summarized that the vast majority of known drug targets, which were mainly composed of small-molecule drug targets, belong to biochemical groups including enzymes, GPCRs, ion channels, transporters, kinases, and NHRs [21], [32]. However, we observed that protein drug targets are not typically enzymes, GPCRs, ion channels, kinase, or NHRs and even significantly lack transporters (Table 2), suggesting a potential great difference from small-molecule drug targets.

Finally in terms of pathway and network analyses, compared with other proteins, we first observed that protein drug targets are involved in significantly fewer metabolic reactions (Table 1). This indicates again that unlike small-molecule drugs, protein drugs do not tend to intervene in metabolic processes. Furthermore, protein drug targets tend to participate in more biological pathways and topologically occupy more important positions in the PPI and signal transduction networks (Table 1). Finally, in the transcriptional regulation network, we found that protein drug targets are regulated by significantly more TFs (Table 1), which is consistent with their higher tissue specificity, since a high level of transcriptional regulation may be needed for tissue-specific genes [59].

For the second question, we observed that successful protein drug targets are significantly different from those of conventional small-molecule drugs in many aspects, including AA composition and physicochemical properties, protein structure, evolution, network topology, and especially function (Tables S4 and S5). For example, evolutionarily, protein drug targets tend to be significantly younger, with higher evolutionary rate, and more nonsynonymously polymorphic in the human population (Table S4). In terms of function, the two types of drug targets have significantly different biochemical distributions. Protein drug targets have significantly lower proportions of enzymes, GPRCs, ion channels, and transporters than small-molecule drug targets (Table S5).

For peptide drug targets, compared with both other nontarget proteins and small-molecule drug targets, we found that they also have distinctive characteristics (Tables S6–S9). In addition, peptide and protein drug targets are also distinguishable in terms of a number of properties (Tables S10 and S11).

In summary, both successful protein and peptide drug targets have their own specific characteristics (Table S12), and are also significantly different from those of small-molecule drugs in multiple aspects. Therefore, it is necessary to construct target prediction models specifically for protein and peptide drugs of current high interest, and the establishment of efficient prediction models is also feasible based on these distinctive characteristics.

### The models can effectively predict targets of protein and peptide drugs

In this section, we constructed separate target prediction models for protein and peptide drugs.

**Supplementary Figure S2:**
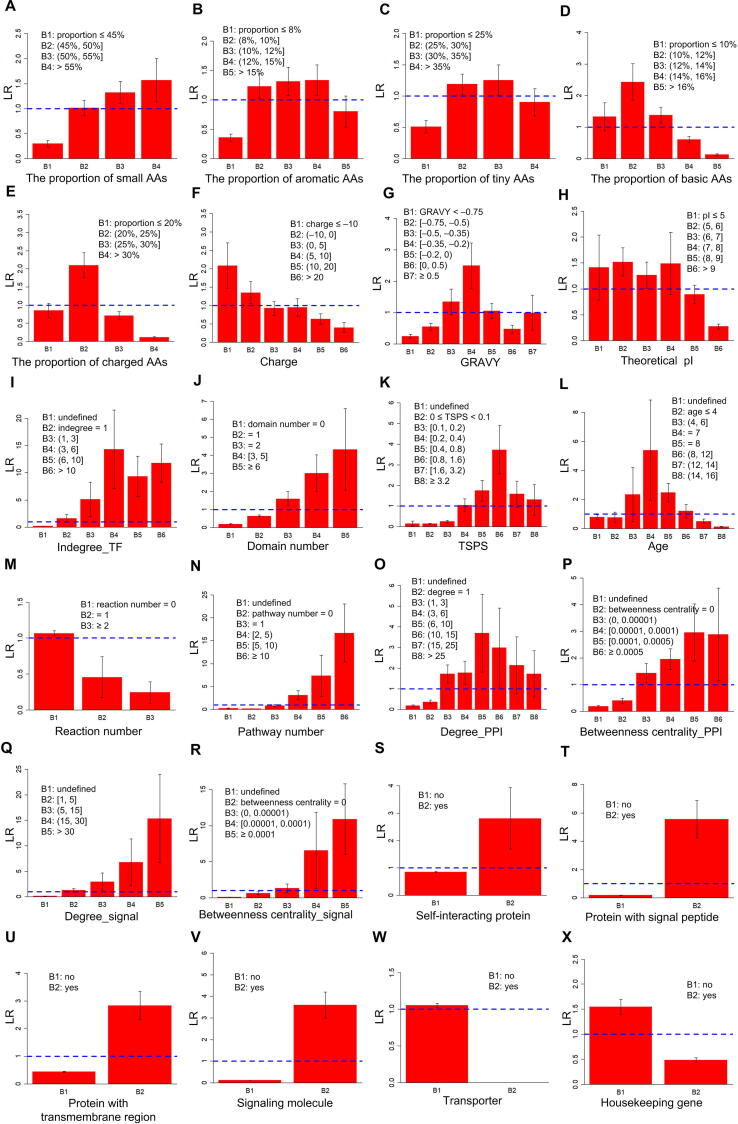
**LRs of various features used for target prediction for protein drugs** The LR of feature f is defined as the ratio of the proportion of proteins satisfying feature f in the GSP set to that in the GSN set. Generally, “LR > 1” means that the feature has some degree of predictive ability, which is marked by the blue dashed line on each graph. The GSN set was repeatedly constructed 100 times (see Method in the main document), and thus the LRs presented in these figures are the mean and SD (denoted by error bar) of these 100 times. **A.–E.** The proportion of small, aromatic, tiny, basic, and charged AAs in the protein sequence. **F.** Charge. **G.** GRAVY (see Method in the main document). **H.** Theoretical pI. **I.** Indegree in the transcriptional regulation network. The indegree of a gene is the number of TFs that regulate the (target) gene. **J.** Domain number. Here, the number of domains (including repeats) rather than the number of domain types of a protein was counted. **K.** TSPS (see Method in the main document). The bin of B1 includes proteins without computed TSPS because of a lack of corresponding gene expression profile data. **L.** Original age. Here, proteins were grouped into 16 age classes, from the oldest “Cellular organisms” class of age 16, composed of proteins that originated from the common ancestor of the three domains of the tree of life (Eukaryota, Bacteria, and Archaea), to the youngest “Homo sapiens” class of age 1, whose proteins are only found in humans. Proteins of age classes from 2 to 15 are, respectively, those that originated from the ancestor of Homininae, Euarchontoglires, Eutheria, Theria, Mammalia, Amniota, Euteleostomi, Chordata, Coelomata, Bilateria, Eumetazoa, Metazoa, the Fungi/Metazoa group, and Eukaryota (please refer to our previous work [44] for more details). **M.** and **N.** Number of reactions and pathways, respectively, in which a protein participates. **O.** and **P.** Degree and betweenness centrality in the PPI network. **Q.** and **R.** Degree and betweenness centrality in the signal transduction network. **S.** Self-interacting protein. **T.** Protein with signal peptide. U. Protein with transmembrane region. **V.** Signaling molecule. **W.** Transporter. **X.** Housekeeping gene.

**Supplementary Figure S3:**
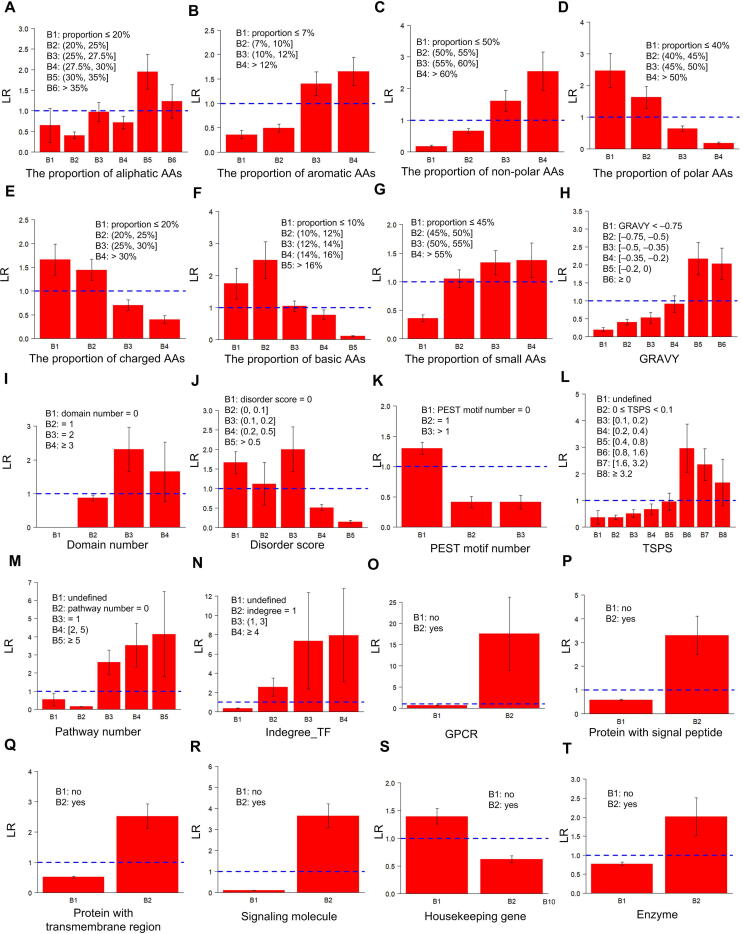
**LRs of various features used for target prediction for peptide drugs** Generally, “LR > 1” means that the feature has some degree of predictive ability, which is marked by the blue dashed line on each graph. The GSN set was repeatedly constructed 100 times, and thus LRs presented in these figures are the mean and SD (denoted by error bar) of the 100 times. **A.–G.** The proportion of aliphatic, aromatic, nonpolar, polar, charged, basic, and small AAs in the protein sequence. **H.** GRAVY**. I.** Domain number. Here the number of domains (including repeats) rather than the number of domain types of a protein was counted. **J.** Disorder score. **K.** PEST motif number. **L.** TSPS. **M.** Number of pathways in which a protein participates. **N.** Indegree in the transcriptional regulation network. The indegree of a gene is the number of TFs regulating the (target) gene. **O.** GPCR. **P.** Protein with signal peptide. **Q.** Protein with transmembrane region. **R.** Signaling molecule. **S.** Housekeeping gene. **T.** Enzyme. PEST, a peptide sequence enriched in proline (P), glutamic acid (E), serine (S), and threonine (T); GPCR, G protein-coupled receptor.

To build the prediction models, efficient prediction features were first collected. Theoretically, all statistically significant features of the protein/peptide drug targets revealed above can be efficiently used for prediction, which was confirmed by the LR results (Figures S2 and S3; see Method).

These collected features were confirmed to be efficient; however, their prediction abilities are different, and there is some redundancy between them (Table S13). It has been recognized that the integration of all individually efficient features is not necessary to achieve the best prediction performance, and the redundancy between features often has no positive or sometimes even a negative impact on the classification performance [51], [60]. Therefore, we used the mRMR method to rank these features based on both efficiency and redundancy (see Method). For the features for the target prediction of protein drugs (Table S14 and Table S15), we observed that the features ranked at the bottom were either those with low efficiency (*e.g.*, “Aromatic”) (Table S16) or those that have relatively high redundancy with the features ranked at the top. For example, the feature “Degree_signal”, with relatively high prediction ability (Table S16), was ranked low mainly because of its high redundancy with “Betweenness centrality_signal” (Table S13), which was ranked at the top.

**Figure 3:**
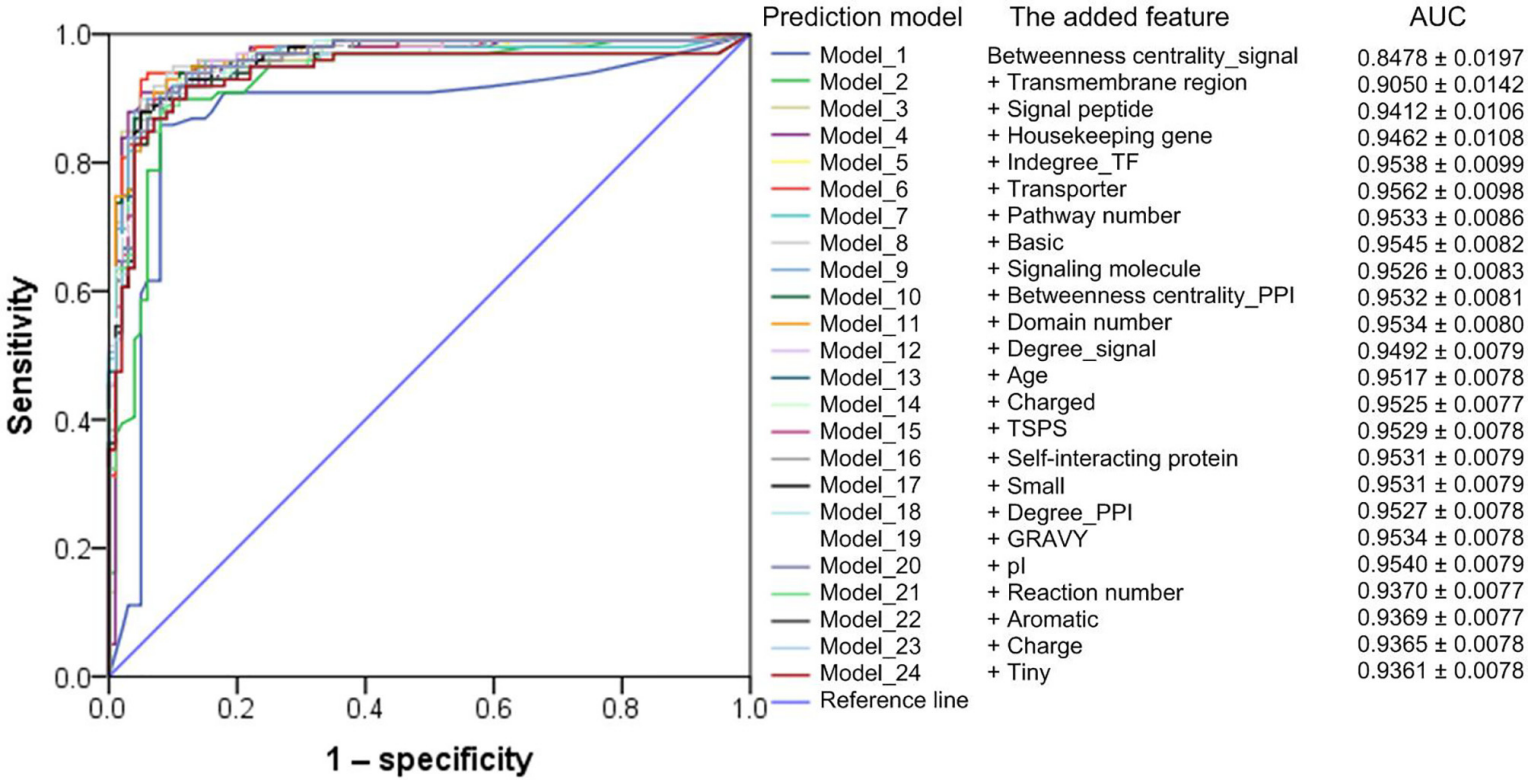
**Performance of the protein drug target prediction models based on 10-fold cross-validation** Starting from the “Betweenness centrality_signal” single-feature prediction model (“Model_1”), the features were introduced into the model one by one according to the order given by mRMR method (Table S14), and the final “Model_24” integrated all 24 features. ROC curves of these models were plotted in different colors. The GSN set was repeatedly constructed 100 times, and thus the mean ± SD of the ROC AUCs of the 100 times for each prediction model is given in the figure. The ROC curves in the figure were drawn based on the GSP set and a random GSN set. ROC, receiver operating characteristic; AUC, area under the curve; GSP, gold standard positive; GSN, gold standard negative; SD, standard deviation; GRAVY, the grand average of hydropathy; pI, isoelectric point; PPI, protein–protein interaction; TSPS, the tissue specificity score.

**Supplementary Figure S4:**
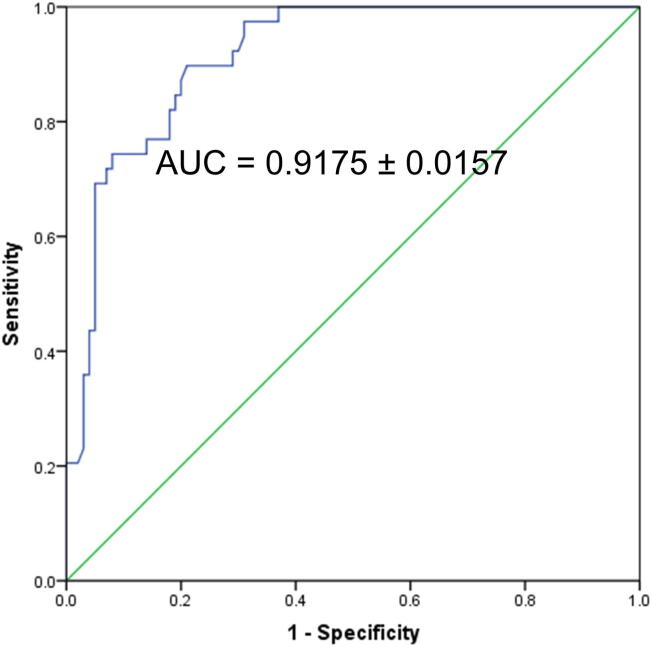
**Performance of “Model_6_peptide” based on 10-fold cross-validation** “Model_6_peptide” is the target prediction model for peptide drugs, integrating six features, including “Signaling molecule”, “Housekeeping gene”, “Non-polar”, “Indegree_TF”, “GPCR” and “Signal peptide”. The GSN set was repeatedly constructed 100 times; thus, the mean ± SD of AUCs of the 100 times is presented in the figure. The ROC curve in the figure was drawn based on the GSP and a random GSN.

Further, we adopted the naïve Bayes rule to integrate these features to construct the target prediction model. For the model construction for protein drugs, starting from the most powerful feature, “Betweenness centrality_signal”, according to the feature order ranked by mRMR (Table S14), we added features into the integrated prediction model one by one. Ultimately, 24 models were obtained (Figure 3), ranging from Model_1 (which was only composed of “Betweenness centrality_signal”) to Model_24 (integrating all features). From the 10-fold cross-validation results, we observed that, as we expected, integrating more features into the model did not lead to stronger prediction efficacy. By and large, the area under the curve (AUC) was gradually improved as the features were added one by one, but we observed that after six features were integrated, the performance remained unchanged or even declined (Figure 3). This again confirmed that good classification performance can be obtained only by integrating several “representative” features with relatively high efficacy and low redundancy [51]. Finally, we took the “Model_6_protein”, which integrated six features, including “Betweenness centrality_signal”, “Transmembrane region”, “Signal peptide”, “Housekeeping gene”, “Indegree_TF”, and “Transporter”, as the final prediction model of protein drug targets, which achieved an outstanding AUC of 0.9562 ± 0.0098. Similarly, we established a model for peptide drugs, “Model_6_peptide”, integrating “Signaling molecule”, “Housekeeping gene”, “Non-polar”, “Indegree_TF”, “GPCR”, and “Signal peptide”, whose AUC reached 0.9175 ± 0.0157, indicating excellent prediction ability (Figure S4).

**Figure 4:**
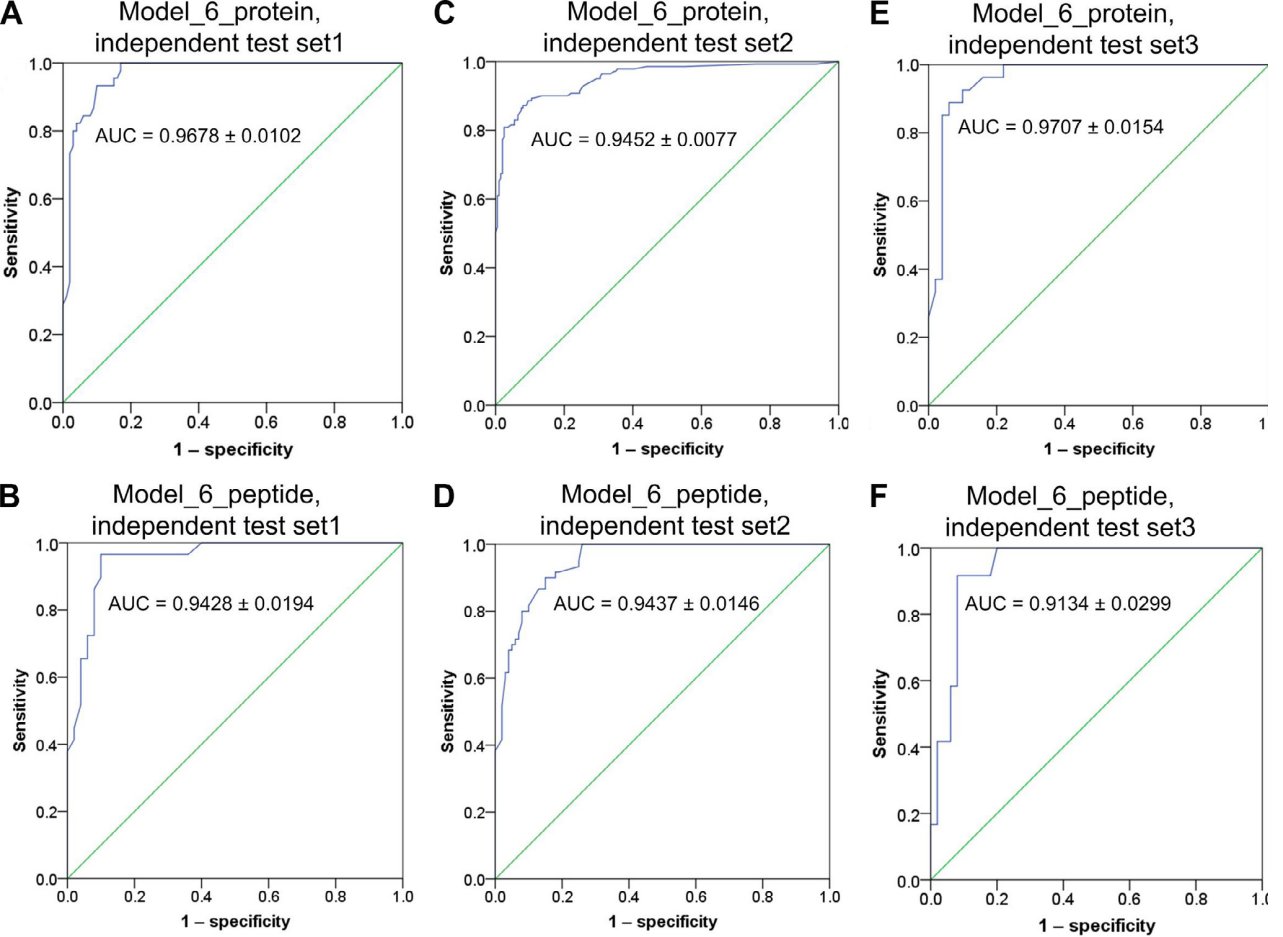
**Performance of “Model_6_protein” and “Model_6_peptide” based on independent test sets** Both the GSN set and the independent negative test set were repeatedly constructed 100 times, and thus, the given AUC on each of these subfigures is the mean ± SD of the 10,000 (= 100 × 100) times. The ROC curves were drawn based on a random GSN set and a random independent negative test set. The green diagonal line on each subfigure is the reference line of the ROC curve plot, with AUC of 0.5. **A.** and **B.** ROC curves and AUCs of “Model_6_protein” (A) and “Model_6_peptide” (B), respectively, using approved protein and peptide drug targets newly recorded by the latest version of DrugBank (downloaded on July 3, 2018) as the independent positive test sets. **C.** and **D.** ROC curves and AUCs of “Model_6_protein” (C) and “Model_6_peptide” (D), respectively, using the targets of clinical trial protein and peptide drugs as the independent positive test sets. **E.** and **F.** ROC curves and AUCs of “Model_6_protein” (E) and “Model_6_peptide” (F), respectively, which were constructed based on the targets of drugs approved before 2010 as the positive training sets and the targets of drugs approved in or after 2010 as the independent positive test sets. See Method for more details.

In addition to the 10-fold cross-validation, independent test schemes were also used to evaluate the performance of the prediction models. Here, we used three independent test datasets. First, we used the therapeutic target set of approved protein/peptide drugs newly added by the latest version of DrugBank (released on July 3, 2018) as the independent positive test set (see Method). Against this independent test set, “Model_6_protein” achieved an AUC of 0.9678 ± 0.0102 and “Model_6_peptide” achieved an AUC of 0.9428 ± 0.0194, validating the high discriminative efficacy of these two models (Figure 4A and B). Furthermore, we applied the prediction models to targets of clinical trial protein and peptide drugs collected by Rask-Andersen et al. [29] and obtained satisfactory performance (Figure 4C and D). In the third test, the GSP set was divided into two subsets. One was composed of therapeutic targets of drugs approved before 2010, and the other included new targets brought by drugs approved in or after 2010 (see Method). The former was used to train the parameters of “Model_6_protein”/“Model_6_peptide”, and the latter was used as the independent positive test set. This scheme was designed to stimulate the real development process of drug targets, to approximately estimate the performance of our prediction system on future truly successful targets. The results showed that when “Model_6_protein” and “Model_6_peptide” were trained using this scheme, they still yielded satisfactory performance against the corresponding independent test sets (Figure 4E and F), strongly showing the effectiveness of our prediction system for distinguishing true targets from other nontarget proteins.

Finally, to further investigate the effect of homologous proteins on the prediction performance of the two prediction models (“Model_6_protein” and “Model_6_peptide”), we adopted three additional types of independent test sets as follows. For the first type, we removed homologous redundancy from the original independent test positive and negative sets (as described above), respectively. Against such three non-redundant independent test sets, both “Model_6_protein” and “Model_6_peptide” still obtained good performance (Table S17). For the second type, from each of the original independent test sets, we excluded proteins homologous with those in the gold standard dataset. Against such three independent test sets, the two prediction models still obtained good performance, indicating that these two models have good prediction efficacy on “*de novo* targets” (Table S18). For the third type, keeping the original independent test positive set unchanged, we re-constructed the independent test negative set and ensured that each protein in the independent test negative set was homologous with at least one protein in the independent test positive set. Against such three independent test sets, the two models also achieved good performance (Table S19), indicating that our prediction models can well distinguish the real targets and nontargets even among a group of homologous proteins.

### A practical web server is established for protein/peptide drug target prediction

**Figure 5:**
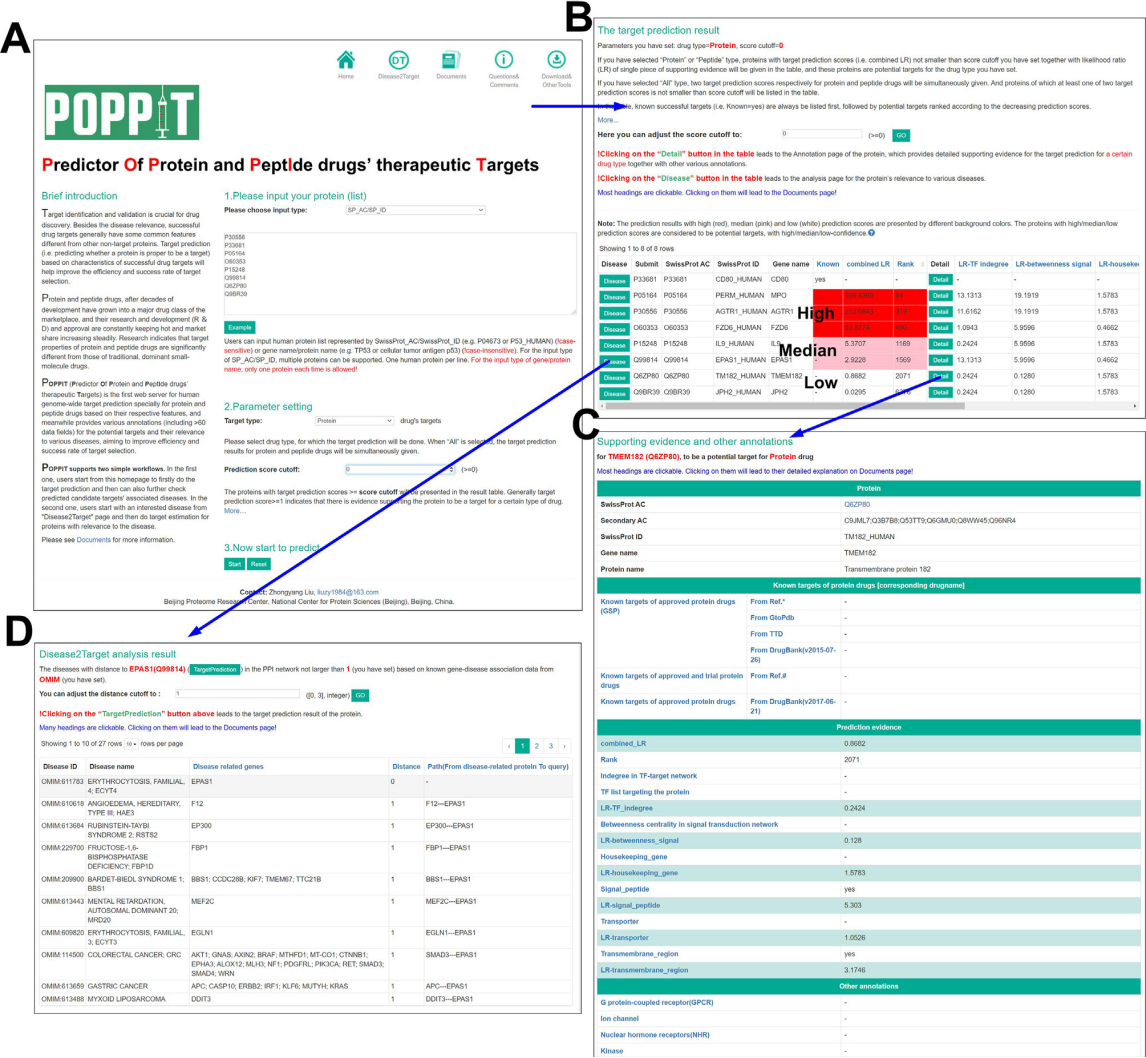
**POPPIT web server** **A.** Homepage of POPPIT. On this page, users can submit a group of proteins of interest, and specify the drug type for which the target prediction should be performed and the score cutoff (only proteins with target prediction scores not smaller than this cutoff will be presented in the result table). **B.** Target prediction result page. On this page, POPPIT will give the target prediction scores *(i.e.*, combined_LRs) of the user-submitted proteins for the user-specified drug type, together with every feature’s respective LR in the prediction model. Only the submitted proteins that are known targets or with prediction scores not smaller than user-specified cutoff (which can be adjusted on this page) will be presented in the result table. The prediction results are grouped into high (represented by a red background), median (pink), and low (white) confidence grades according to the prediction scores. The column “Known” tells us whether this protein belongs to the GSP set of the user-specified drug type, and “Rank” was obtained based on the whole genome ranked according to decreasing prediction scores. Clicking on the “Detail” button in the table will lead to the annotation page of this candidate target protein, and the “Disease” button to the “Disease relevance analysis” page. **C.** Annotation page of a candidate target protein. This page gives a protein’s detailed supporting evidence for being a target of a certain drug type and other various annotations. **D.** “Disease relevance analysis” result page. This page presents the list of diseases relevant to a candidate target protein. In POPPIT the relevance between a disease and a protein was measured by their distance in the PPI network [58], defined as the minimum shortest path length between the known related genes of the disease and the protein. In the result table, for each associated disease, we present its known related genes, its distance to the protein of interest, and the corresponding path in the PPI network. Only diseases with distances to the protein not larger than the user-specified cutoff will be listed in the result table. This cutoff can also be adjusted on this page.

For ease of use of our target prediction methods, we developed a user-friendly web server named POPPIT. The purpose of this web server was to perform genome-wide target prediction for protein and peptide drugs, and to also provide abundant annotations for the predicted targets together with their relevance to various diseases. POPPIT aims to prioritize candidate targets and increase the efficiency and success rate of target selection during drug discovery for protein and peptide drugs (Figure 5A).

In POPPIT, for a protein of interest, a target prediction score for the protein/peptide drug is given by “Model_6_protein”/“Model_6_peptide” (Figure 5B). Each candidate target protein’s annotation page contains > 60 categories of annotations, involving its corresponding approved/trial drugs (if the candidate target protein has those), detailed supporting evidence for its target prediction, and various properties and information about physicochemistry, function, tissue specificity, evolution, protein structure, biological pathways and networks (Figure 5C). In addition, for each potential target, POPPIT also provides a “Disease relevance analysis” function, by which users can check various diseases to which the candidate target protein is relevant (Figure 5D). This relevance between a protein and a disease was quantified by their distance in the PPI network [58], defined as the minimum shortest path length between the protein and the known related genes of the disease (File S1). A distance of zero means that the protein happens to be the disease’s known related gene. In addition to the protein of interest, on the “Disease2Target” page of the POPPIT website, users can start with a disease of interest and then perform target prediction for proteins relevant to this disease.

With POPPIT, we found that some proteins with high target prediction scores can be validated by recently approved drugs that target these proteins (Table S20). For example, the alpha subunit of interleukin-5 receptor (gene name: *IL5RA*, combined_LR = 7049, rank = 3) has been a successful therapeutic target of a protein drug. It is specifically bound by benralizumab, which is a humanized recombinant monoclonal antibody and was recently approved by the FDA, as a maintenance treatment for patients older than 12 years of age with severe eosinophilic asthma (https://www.accessdata.fda.gov/drugsatfda_docs/label/2017/761070s000lbl.pdf). Meanwhile, according to the “Disease relevance analysis” function of POPPIT, we found that the alpha subunit of interleukin-5 receptor is related to asthma (distance = 1) by its direct interaction with interleukin-5 (gene name: *IL5*), which is known to play a role in the etiology of asthma [61]. This association with asthma in the PPI network is also consistent with benralizumab’s MoA, that is, benralizumab inhibits the binding between interleukin-5 and the alpha subunit of interleukin-5 receptor and thus blocks the interleukin-5-induced eosinophil-mediated inflammatory response [61]. Other examples include B-cell receptor CD22 (gene name: *CD22*), interleukin-4 receptor subunit alpha (gene name: *IL4R*), leptin receptor (gene name: *LEPR*), and melanocortin receptor 4 (gene name: *MC4R*), whose corresponding drugs have also been approved by the FDA recently (Table S20). These recently successfully validated targets show that POPPIT can effectively support target identification.

## Discussion

In the field of drug discovery, the systematic exploration of target space in the human genome has always drawn high attention [16], [17], [21], [23], [30], [62]. However, because relative to small-molecule drugs, the development of protein/peptide drugs started late, studies specifically on protein and peptide drugs of current high interest are still lacking. Here, we systematically explored the target spaces of protein and peptide drugs. We systematically presented the distinctive characteristics of the targets of protein and peptide drugs from multiple aspects and described the differences between targets of protein, peptide, and small-molecule drugs. Then, the first effective genome-wide target prediction model of protein/peptide drugs was constructed. Finally, a practical web server called POPPIT was developed, providing target prediction for protein/peptide drugs and various annotations for candidate target proteins.

Here we found that protein/peptide drug targets have many distinctive characteristics. However, we should be cautious that all the properties have certain correlations between each other (Table S13). For example, protein drug targets tend to be hydrophobic compared with nontarget proteins (Table 1, mean of GRAVY: −0.2596 > −0.3324, rank sum test, *P* = 0.0226), but after controlling the “transmembrane region” factor, protein drug targets inversely tend to be hydrophilic compared with other proteins (mean of GRAVY: −0.2596 < −0.1264, rank sum test, *P* = 0.0001). The “transmembrane region” factor control was implemented by constructing a negative control set with a (approximately) same distribution as the positive target set on “transmembrane region” property. This result suggests that the characteristic of higher hydrophobicity of protein drug targets may be related to their higher probability of containing transmembrane regions, and indeed the “transmembrane region” property has a positive correlation with the “hydrophobicity” property (Table S13, Spearman rank correlation coefficient is 0.51). Therefore, during the target prediction model construction, we have considered the correlation (*i.e*., redundancy) between different features and performed mRMR feature selection (see Method).

The aim of the constructed protein/peptide drug target prediction model and the corresponding web server POPPIT is to predict proper protein/peptide drug targets from disease-related proteins, improving the efficiency and success rate of the “target identification” step in a “target-based drug discovery” strategy [63]. Specifically, various disease-related studies, especially disease-related omics researches, are revealing a large number of disease-related proteins, bringing unprecedented opportunities for target discovery [64]. However, it is still a challenging problem to decide which disease-related proteins are more likely to be drug targets for further experimental validation. Currently researchers have to manually select candidate protein/peptide drug targets from disease-related proteins only based on the limited experience. For example, in order to nominate candidate targets from proteomics study-derived gastric cancer-related proteins, Ge et al*.* simply considered the extracellular membrane proteins among them as candidate targets for protein drugs [65]. Sanseau et al., from GWAS genes associated with various diseases, directly selected the protein products with either a signal peptide or transmembrane domain as the candidate targets for protein drugs, as an approximation [26]. Currently there is no bioinformatics method and tool, which can systematically consider various features learned from the successful targets, to help select promising candidate targets for protein/peptide drugs. Our work can resolve this problem. POPPIT will benefit target discovery, especially current omics-based target discovery. Of course once a disease-associated protein is predicted as a target, various *in vitro* and *in vivo* experiments should be designed to further validate this potential target. Then, for a validated candidate target, candidate drugs can be screened or designed [63].

**Supplementary Figure S5:**
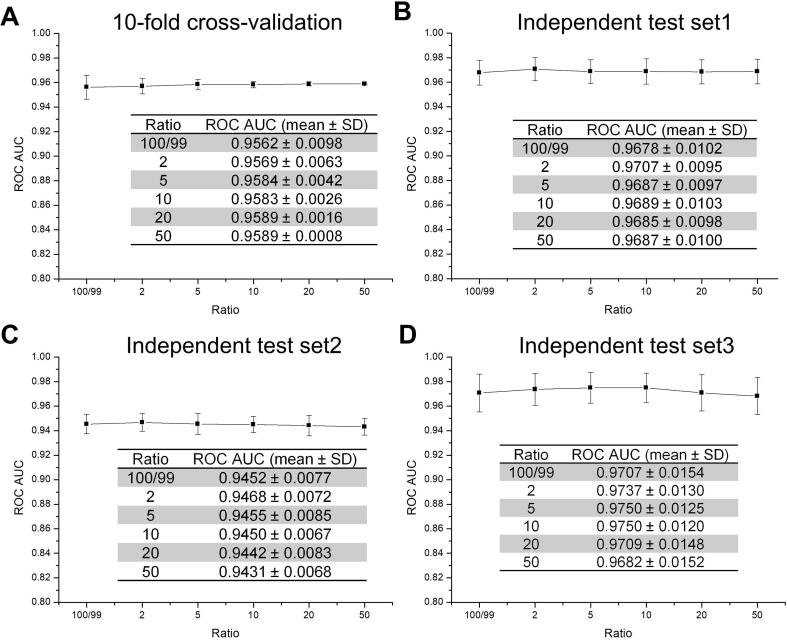
**ROC AUCs of “Model_6_protein” across different ratios of the GSN set size to the GSP set size** Here the size of the GSP set was kept unchanged (size = 99 used in the main document). The size of the GSN set was, respectively, set to 2×, 5×, 10×, 20×, 50×, and 100/99× (used in the main document) the size of the GSP set, and the GSN set was constructed using the same method as described in the main document. The protein drug target prediction models (i.e. “Model_6_protein”) were constructed based on the GSP set and the GSN sets with different sizes, respectively. **A.** ROC AUCs based on 10-fold cross-validation. **B.** ROC AUCs based on the first independent test set. The first independent test positive set was composed of the newly added therapeutic target set of the approved protein drugs from the latest version of DrugBank (released on July 3, 2018). **C.** ROC AUCs based on the second independent test set. The second independent test positive set was composed of targets of clinical trial protein drugs [29]. **D.** ROC AUCs based on the third independent test set. For the third independent test set, we divided the GSP set into two parts. One part was composed of targets of protein drugs approved before 2010, which was used as the positive training set; the other was composed of the targets of protein drugs approved in or after 2010, which was used as the independent positive test set. See more details of the three independent test sets in Method of the main document. For the 10-fold cross-validation (A), for each ratio, the GSN set was repeatedly constructed 100 times, and thus the shown AUC is the mean ± SD of the results of 100 times. For the independent tests (B–D), the GSN set and the independent test negative set were repeatedly constructed 10 times, respectively, and thus the AUC is the mean ± SD of the results of 100 (= 10 × 10) times.

Generally, the positive and negative sets of drug targets should be very imbalanced. However, it is difficult to estimate the real ratio between the positive and negative set sizes. Fortunately, we find that the impact of the ratio is very small on the prediction ability of the constructed prediction model. ROC AUCs of “Model_6_protein” almost remained unchanged across different ratios against both 10-fold cross-validation and three independent test sets (Figure S5).

Naïve Bayes was adopted to establish the prediction models mainly based on the following considerations. On the one hand, for this work, the performance of naïve Bayes was better than other shallow machine learning methods, including the support vector machine (SVM), neural network (NN), decision tree (DT), random forest (RF), logistic regression (LR), and K-nearest neighbor (KNN) (File S1; Table S21). On the other hand, naïve Bayes has several commendable advantages such as good interpretability, small sample size requirement, and low computational resource consumption.

In summary, this study performed a systematic exploration of target spaces in the human genome for protein and peptide drugs. It not only contributes to the understanding of the distinctive cellular roles of the targets and the MoA of protein/peptide drugs from an overall perspective, but also, more importantly, provides algorithms and a practical tool for performing quantitative target prediction, together with lengthy annotations. This work will help improve the efficiency and success rate of target selection, and ultimately contribute to novel target discovery for protein and peptide drugs.

## Data availability

POPPIT is available at http://poppit.ncpsb.org.cn/.

## CRediT author statement

**Zhongyang Liu:** Conceptualization, Methodology, Software, Validation, Formal analysis, Writing - original draft, Writing - review & editing, Visualization, Funding acquisition. **Honglei Li:** Software. **Zhaoyu Jin:** Writing - review & editing. **Yang Li:** Investigation. **Feifei Guo:** Investigation. **Yangzhige He:** Investigation. **Xinyue Liu:** Writing - review & editing. **Yaning Qi:** Writing - review & editing. **Liying Yuan:** Investigation. **Fuchu He:** Supervision, Funding acquisition. **Dong Li:** Supervision, Funding acquisition, Writing - review & editing. All authors have read and approved the final manuscript.

## Competing interests

The authors have declared no competing interests.

## Figures and Tables

**Supplementary Figure S1:**
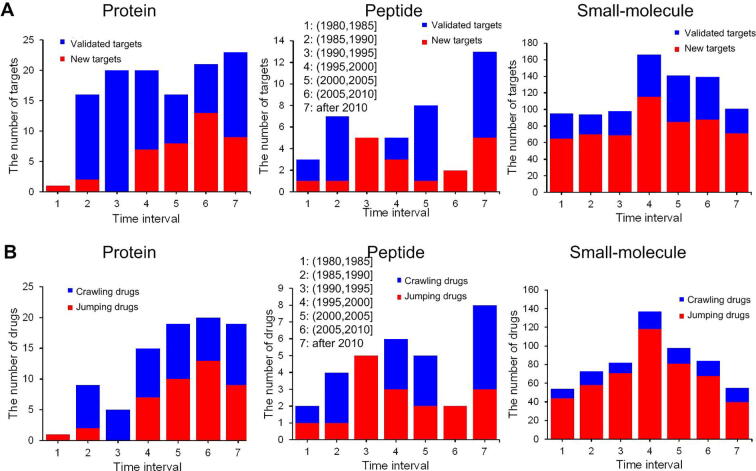
**The composition of new drugs approved every five years and their targets** The results were obtained based on the therapeutic target data of approved drugs from DrugBank (downloaded on July 26, 2015) (see Method in the main document). **A.** The composition of the targets of new drugs approved every five years. “Validated targets” refer to those that have been used as targets of previously approved drugs, while “new targets” have not. A bin is five years, and the year scope of each bin is described on the graph. The average proportion of “new targets” for protein drugs is significantly larger than that for small-molecule drugs (57.35% > 31.93%, rank sum test, P value = 0.0380), while there is no statistically significant difference between the proportions for peptide and small-molecule drugs (48.77% > 31.93%, P value = 0.2090). **B.** The composition of new drugs approved every five years. “Jumping drugs” are those with totally new target sets, whereas “crawling drugs” have at least one “validated target” [58]. The average percentage of “jumping drugs” among protein drugs is significantly larger than that among small-molecule drugs (52.30% > 18.57%, rank sum test, P value = 0.0260), while there was no statistically significant difference between the proportions of “jumping” peptide and small-molecule drugs (42.50% > 18.57%, P value = 0.2090).
