# Supplementary material for: Exploration of Target Spaces in the Human Genome for Protein and Peptide Drugs
Source: Genomics Proteomics Bioinformatics. 2022 Mar 23;20(4):780–94. doi: 10.1016/j.gpb.2021.10.007 (PMC9881050; doi:10.1016/j.gpb.2021.10.007)
Supplement: Supplementary Table S4 [file mmc4.docx]

**Table S4 Quantitative differences between protein and small-molecule drug targets**

| Property | Mean value (mean rank) | | *P* value  (rank sum test,  one-sided) ^1^ | Adjusted  *P* value ^1^ |
| --- | --- | --- | --- | --- |
|  | **Protein drug**  **targets** | **Small-molecule**  **drug targets** |  |  |
| Tiny (%) | 29.9664 (257) | 28.7872 (222) | **9.50E–03** | **1.28E–02** |
| Small (%) | 51.6968 (276) | 49.4576 (217) | **4.24E–05** | **1.43E–04** |
| Aliphatic (%) | 27.5851 (171) | 29.9875 (246) | **3.50E–07** | **3.15E–06** |
| Aromatic (%) | 10.8935 (194) | 11.7626 (239) | **1.30E–03** | **2.34E–03** |
| Non-polar (%) | 54.4097 (192) | 56.1500 (240) | **7.67E–04** | **1.59E–03** |
| Polar (%) | 45.5903 (267) | 43.8500 (219) | **7.67E–04** | **1.59E–03** |
| Charged (%) | 22.9411 (209) | 23.7356 (235) | **3.97E–02** | **4.46E–02** |
| Basic (%) | 12.0871 (192) | 12.9825 (240) | **6.27E–04** | **1.54E–03** |
| Acidic (%) | 10.8539 (232) | 10.7531 (229) | 4.23E–01 | 4.23E–01 |
| GRAVY | –0.2596 (187) | –0.1429 (241) | **1.50E–04** | **4.50E–04** |
| Theoretical pI | 6.5817 (188) | 7.1406 (241) | **2.41E–04** | **6.49E–04** |
| Charge | –2.4444 (180) | 3.8886 (243) | **1.36E–05** | **5.25E–05** |
| Domain number | 3.3333 (255) | 2.1560 (223) | **1.23E–02** | **1.59E–02** |
| Disorder score | 0.1799 (263) | 0.1380 (220) | **1.71E–03** | **2.89E–03** |
| PEST motif number | 0.6364 (248) | 0.5042 (224) | **2.48E–02** | **3.05E–02** |
| TSPS | 1.3393 (249) | 1.2994 (222) | **3.62E–02** | **4.25E–02** |
| Age | 8.5500 (108) | 11.7034 (207) | **5.69E–14** | **1.54E–12** |
| Evolutionary rate | 5.5031 (224) | 1.6763 (162) | **1.01E–06** | **5.45E–06** |
| *C_ratio_* | 38.5784 (235) | 19.2538 (196) | **3.34E–03** | **5.30E–03** |
| Pathway number | 6.1327 (264) | 5.0391 (219) | **1.21E–03** | **2.34E–03** |
| Reaction number | 0.0404 (178) | 1.4875 (244) | **3.05E–09** | **4.12E–08** |
| Degree_PPI | 9.9565 (200) | 17.3871 (181) | 6.71E–02 | 7.24E–02 |
| Betweenness centrality_PPI | 0.0002 (209) | 0.0011 (178) | **8.40E–03** | **1.19E–02** |
| Degree_signal | 40.8778 (211) | 27.3008 (153) | **7.12E–07** | **4.81E–06** |
| Betweenness centrality_signal | 0.0012 (205) | 0.0009 (155) | **1.20E–05** | **5.25E–05** |
| Indegree_TF | 7.4815 (174) | 6.5000 (145) | **5.24E–03** | **7.85E–03** |
| Outdegree_TF | 4.0000 (17) | 26.1724 (19) | 3.27E–01 | 3.40E–01 |

*Note*: ^1^, *P* values smaller than 0.05 are represented in bold type. Adjusted *P* value was computed by Benjamini-Hochberg multiple testing correction method.
