# Supplementary material for: Exploration of Target Spaces in the Human Genome for Protein and Peptide Drugs
Source: Genomics Proteomics Bioinformatics. 2022 Mar 23;20(4):780–94. doi: 10.1016/j.gpb.2021.10.007 (PMC9881050; doi:10.1016/j.gpb.2021.10.007)
Supplement: Supplementary Table S5 [file mmc5.docx]

**Table S5 Qualitative differences between protein and small-molecule drug targets**

| Property | The fraction of proteins belonging  to a certain protein class (%) | | *P* value  (Fisher’s exact test,  one-sided) ^1^ | Adjusted  *P* value ^1^ |
| --- | --- | --- | --- | --- |
|  | **Protein drug**  **targets** | **Small-molecule**  **drug targets** |  |  |
| Protein with signal peptide | 84.85 | 25.07 | **1.50E–27** | **1.80E–26** |
| Protein with transmembrane region | 66.67 | 47.91 | **6.46E–04** | **1.29E–03** |
| Signaling molecule | 90.91 | 68.52 | **1.55E–06** | **4.65E–06** |
| Transcription factor | 0.00 | 3.34 | 5.17E–02 | 6.89E–02 |
| Housekeeping gene | 24.24 | 37.05 | **1.09E–02** | **1.63E–02** |
| Self-interacting protein | 22.22 | 20.06 | 3.65E–01 | 3.65E–01 |
| Enzyme | 15.15 | 47.08 | **1.85E–09** | **1.11E–08** |
| GPCR | 5.05 | 13.65 | **1.05E–02** | **1.63E–02** |
| Ion channel | 0.00 | 11.98 | **1.58E–05** | **3.78E–05** |
| NHR | 0.00 | 1.95 | 1.79E–01 | 2.15E–01 |
| Kinase | 3.03 | 4.74 | 3.39E–01 | 3.65E–01 |
| Transporter | 0.00 | 18.94 | **1.37E–08** | **5.47E–08** |

*Note*: ^1^, *P* values smaller than 0.05 are represented in bold type. Adjusted *P* value was computed by Benjamini-Hochberg multiple testing correction method.
