# Supplementary material for: Exploration of Target Spaces in the Human Genome for Protein and Peptide Drugs
Source: Genomics Proteomics Bioinformatics. 2022 Mar 23;20(4):780–94. doi: 10.1016/j.gpb.2021.10.007 (PMC9881050; doi:10.1016/j.gpb.2021.10.007)
Supplement: Supplementary Table S6 [file mmc6.docx]

**Table S6 Quantitative differences between peptide drug targets and other proteins**

| Property | Mean value (mean rank) | | *P* value  (rank sum test,  one-sided) ^1^ | Adjusted  *P* value ^1^ |
| --- | --- | --- | --- | --- |
|  | **Peptide drug targets** | **Other proteins** |  |  |
| Tiny (%) | 29.8808 (5187) | 29.2440 (4523) | 5.65E–02 | 8.77E–02 |
| Small (%) | 50.9714 (5386) | 49.4483 (4522) | **1.96E–02** | **3.78E–02** |
| Aliphatic (%) | 30.2277 (5723) | 28.1231 (4520) | **2.07E–03** | **5.58E–03** |
| Aromatic (%) | 11.4056 (5638) | 10.3888 (4521) | **3.86E–03** | **9.47E–03** |
| Non-polar (%) | 57.4749 (6394) | 53.4345 (4517) | **3.82E–06** | **3.44E–05** |
| Polar (%) | 42.5251 (2657) | 46.5655 (4534) | **3.82E–06** | **3.44E–05** |
| Charged (%) | 23.2723 (3159) | 25.4789 (4531) | **5.29E–04** | **1.59E–03** |
| Basic (%) | 12.0774 (2847) | 14.1400 (4533) | **2.90E–05** | **1.57E–04** |
| Acidic (%) | 11.1949 (3880) | 11.3389 (4528) | 6.11E–02 | 8.77E–02 |
| GRAVY | –0.0979 (6364) | –0.3335 (4517) | **5.26E–06** | **3.55E–05** |
| Theoretical pI | 7.0990 (4182) | 7.3438 (4526) | 2.05E–01 | 2.21E–01 |
| Charge | 1.7949 (4164) | 4.0446 (4527) | 1.94E–01 | 2.18E–01 |
| Domain number | 1.8205 (5864) | 1.4371 (4520) | **1.98E–04** | **7.62E–04** |
| Disorder score | 0.1160 (3045) | 0.2538 (4532) | **1.66E–04** | **7.47E–04** |
| PEST motif number | 0.1795 (3632) | 0.6242 (4529) | **4.74E–03** | **1.07E–02** |
| TSPS | 1.5738 (5501) | 0.9548 (4154) | **2.83E–04** | **9.55E–04** |
| Age | 9.9138 (2909) | 10.9305 (3408) | 8.43E–02 | 1.08E–01 |
| Evolutionary rate | 0.3266 (3245) | 4.5235 (3192) | 4.40E–01 | 4.40E–01 |
| *C_ratio_* | 39.3085 (3513) | 34.3793 (3627) | 3.76E–01 | 3.90E–01 |
| Pathway number | 3.2105 (6787) | 1.0137 (4252) | **1.08E–14** | **2.92E–13** |
| Reaction number | 0.3077 (4727) | 0.4428 (4525) | 1.52E–01 | 1.79E–01 |
| Degree_PPI | 8.8889 (3234) | 8.7197 (2818) | 6.17E–02 | 8.77E–02 |
| Betweenness centrality_PPI | 0.0001 (3307) | 0.0001 (2818) | **3.50E–02** | 5.91E–02 |
| Degree_signal | 29.3056 (1358) | 16.3019 (1187) | 6.77E–02 | 9.14E–02 |
| Betweenness centrality_signal | 0.0003 (1396) | 0.0003 (1186) | **3.18E–02** | 5.72E–02 |
| Indegree_TF | 3.8519 (968) | 3.1038 (791) | **1.67E–02** | **3.46E–02** |
| Outdegree_TF | 19.3333 (179) | 9.7294 (129) | 1.19E–01 | 1.45E–01 |

*Note*: ^1^, *P* values smaller than 0.05 are represented in bold type. Adjusted *P* value was computed by Benjamini-Hochberg multiple testing correction method.
