# Supplementary material for: Exploration of Target Spaces in the Human Genome for Protein and Peptide Drugs
Source: Genomics Proteomics Bioinformatics. 2022 Mar 23;20(4):780–94. doi: 10.1016/j.gpb.2021.10.007 (PMC9881050; doi:10.1016/j.gpb.2021.10.007)
Supplement: Supplementary Table S7 [file mmc7.docx]

**Table S7 Qualitative differences between peptide drug targets and other proteins**

| Property | The fraction of proteins belonging  to a certain protein class (%) | | *P* value  (Fisher’s exact test,  one-sided) ^1^ | Adjusted  *P* value ^1^ |
| --- | --- | --- | --- | --- |
|  | **Peptide drug**  **targets** | **Other proteins** |  |  |
| Protein with signal peptide | 51.28 | 15.81 | **3.26E–07** | **1.30E–06** |
| Protein with transmembrane region | 61.54 | 24.67 | **1.22E–06** | **3.67E–06** |
| Signaling molecule | 92.31 | 26.02 | **4.16E–18** | **5.00E–17** |
| Transcription factor | 2.56 | 2.95 | 6.80E–01 | 6.80E–01 |
| Housekeeping gene | 30.77 | 50.38 | **1.06E–02** | **2.12E–02** |
| Self-interacting protein | 17.95 | 8.62 | **4.77E–02** | 7.58E–02 |
| Enzyme | 38.46 | 20.66 | **8.41E–03** | **2.02E–02** |
| GPCR | 28.21 | 1.68 | **4.68E–11** | **2.81E–10** |
| Ion channel | 0.00 | 1.02 | 6.71E–01 | 6.80E–01 |
| NHR | 2.56 | 0.12 | 5.05E–02 | 7.58E–02 |
| Kinase | 0.00 | 1.43 | 5.71E–01 | 6.80E–01 |
| Transporter | 0.00 | 5.42 | 1.15E–01 | 1.53E–01 |

*Note*: ^1^, *P* values smaller than 0.05 are represented in bold type. Adjusted *P* value was computed by Benjamini-Hochberg multiple testing correction method.
