# Supplementary material for: Exploration of Target Spaces in the Human Genome for Protein and Peptide Drugs
Source: Genomics Proteomics Bioinformatics. 2022 Mar 23;20(4):780–94. doi: 10.1016/j.gpb.2021.10.007 (PMC9881050; doi:10.1016/j.gpb.2021.10.007)
Supplement: Supplementary Table S8 [file mmc8.docx]

**Table S8 Quantitative differences between peptide and small-molecule drug targets**

| Property | Mean value (mean rank) | | *P* value  (rank sum test,  one-sided) ^1^ | Adjusted  *P* value ^1^ |
| --- | --- | --- | --- | --- |
|  | **Peptide drug**  **targets** | **Small-molecule**  **drug targets** |  |  |
| Tiny (%) | 29.8808 (234) | 28.7872 (196) | **2.40E–02** | 8.09E–02 |
| Small (%) | 50.9714 (237) | 49.4576 (195) | **1.68E–02** | 6.49E–02 |
| Aliphatic (%) | 30.2277 (213) | 29.9875 (198) | 2.12E–01 | 3.23E–01 |
| Aromatic (%) | 11.4056 (195) | 11.7626 (200) | 4.00E–01 | 4.15E–01 |
| Non-polar (%) | 57.4749 (239) | 56.1500 (195) | **1.13E–02** | 6.12E–02 |
| Polar (%) | 42.5251 (160) | 43.8500 (204) | **1.13E–02** | 6.12E–02 |
| Charged (%) | 23.2723 (171) | 23.7356 (203) | 5.28E–02 | 1.29E–01 |
| Basic (%) | 12.0774 (161) | 12.9825 (204) | **1.40E–02** | 6.29E–02 |
| Acidic (%) | 11.1949 (188) | 10.7531 (201) | 2.51E–01 | 3.23E–01 |
| GRAVY | –0.0979 (227) | –0.1429 (197) | 5.78E–02 | 1.30E–01 |
| Theoretical pI | 7.0990 (195) | 7.1406 (200) | 3.93E–01 | 4.15E–01 |
| Charge | 1.7949 (184) | 3.8886 (201) | 1.92E–01 | 3.23E–01 |
| Domain number | 1.8205 (191) | 2.1560 (200) | 2.93E–01 | 3.47E–01 |
| Disorder score | 0.1160 (175) | 0.1380 (202) | 7.77E–02 | 1.61E–01 |
| PEST motif number | 0.1795 (174) | 0.5042 (202) | **2.95E–02** | 8.84E–02 |
| TSPS | 1.5738 (217) | 1.2994 (195) | 1.32E–01 | 2.37E–01 |
| Age | 9.9138 (121) | 11.7034 (164) | **8.06E–03** | 6.12E–02 |
| Evolutionary rate | 0.3266 (187) | 1.6763 (147) | **1.03E–02** | 6.12E–02 |
| *C_ratio_* | 39.3085 (197) | 19.2538 (175) | 1.15E–01 | 2.22E–01 |
| Reaction number | 0.3077 (165) | 1.4875 (203) | **6.46E–03** | 6.12E–02 |
| Pathway number | 3.2105 (170) | 5.0391 (202) | 5.23E–02 | 1.29E–01 |
| Degree_PPI | 8.8889 (166) | 17.3871 (157) | 2.96E–01 | 3.47E–01 |
| Betweenness centrality_PPI | 0.0001 (168) | 0.0011 (157) | 2.51E–01 | 3.23E–01 |
| Degree_signal | 29.3056 (140) | 27.3008 (142) | 4.48E–01 | 4.48E–01 |
| Betweenness centrality_signal | 0.0003 (133) | 0.0009 (143) | 2.40E–01 | 3.23E–01 |
| Indegree_TF | 3.8519 (116) | 6.5000 (127) | 2.27E–01 | 3.23E–01 |
| Outdegree_TF | 19.3333 (18) | 26.1724 (16) | 3.84E–01 | 4.15E–01 |

*Note*: ^1^, *P* values smaller than 0.05 are represented in bold type. Adjusted *P* value was computed by Benjamini-Hochberg multiple testing correction method.
