# Supplementary material for: Exploration of Target Spaces in the Human Genome for Protein and Peptide Drugs
Source: Genomics Proteomics Bioinformatics. 2022 Mar 23;20(4):780–94. doi: 10.1016/j.gpb.2021.10.007 (PMC9881050; doi:10.1016/j.gpb.2021.10.007)
Supplement: Supplementary Table S9 [file mmc9.docx]

**Table S9 Qualitative differences between peptide and small-molecule drug targets**

| Property | The fraction of proteins belonging  to a certain protein class (%) | | *P* value  (Fisher’s exact test,  one-sided) ^1^ | Adjusted  *P* value ^1^ |
| --- | --- | --- | --- | --- |
|  | **Peptide drug**  **targets** | **Small-molecule**  **drug targets** |  |  |
| Protein with signal peptide | 51.28 | 25.07 | **8.34E–04** | **3.34E–03** |
| Protein with transmembrane region | 61.54 | 47.91 | 7.35E–02 | 1.47E–01 |
| Signaling molecule | 92.31 | 68.52 | **7.18E–04** | **3.34E–03** |
| Transcription factor | 2.56 | 3.34 | 6.30E–01 | 6.30E–01 |
| Housekeeping gene | 30.77 | 37.05 | 2.78E–01 | 3.70E–01 |
| Self-interacting protein | 17.95 | 20.06 | 4.73E–01 | 5.68E–01 |
| Enzyme | 38.46 | 47.08 | 1.96E–01 | 2.95E–01 |
| GPCR | 28.21 | 13.65 | **1.97E–02** | **4.74E–02** |
| Ion channel | 0.00 | 11.98 | **9.08E–03** | **2.73E–02** |
| NHR | 2.56 | 1.95 | 5.65E–01 | 6.17E–01 |
| Kinase | 0.00 | 4.74 | 1.67E–01 | 2.86E–01 |
| Transporter | 0.00 | 18.94 | **4.44E–04** | **3.34E–03** |

*Note*: ^1^, *P* values smaller than 0.05 are represented in bold type. Adjusted *P* value was computed by Benjamini-Hochberg multiple testing correction method.
