# Supplementary material for: Exploration of Target Spaces in the Human Genome for Protein and Peptide Drugs
Source: Genomics Proteomics Bioinformatics. 2022 Mar 23;20(4):780–94. doi: 10.1016/j.gpb.2021.10.007 (PMC9881050; doi:10.1016/j.gpb.2021.10.007)
Supplement: Supplementary Table S10 [file mmc10.docx]

**Table S10 Quantitative differences between protein and peptide drug targets**

| Property | Mean value (mean rank) | | *P* value  (rank sum test,  one-sided) ^1^ | Adjusted  *P* value ^1^ |
| --- | --- | --- | --- | --- |
|  | **Protein drug**  **targets** | **Peptide drug**  **targets** |  |  |
| Tiny (%) | 29.9664 (69) | 29.8808 (72) | 3.50E–01 | 4.09E–01 |
| Small (%) | 51.6968 (71) | 50.9714 (67) | 3.08E–01 | 3.96E–01 |
| Aliphatic (%) | 27.5851 (62) | 30.2277 (88) | **2.55E–04** | **1.38E–03** |
| Aromatic (%) | 10.8935 (66) | 11.4056 (78) | 5.04E–02 | 8.52E–02 |
| Non-polar (%) | 54.4097 (61) | 57.4749 (91) | **3.93E–05** | **5.31E–04** |
| Polar (%) | 45.5903 (78) | 42.5251 (48) | **3.93E–05** | **5.31E–04** |
| Charged (%) | 22.9411 (71) | 23.2723 (66) | 2.46E–01 | 3.45E–01 |
| Basic (%) | 12.0871 (70) | 12.0774 (69) | 4.76E–01 | 4.76E–01 |
| Acidic (%) | 10.8539 (71) | 11.1949 (65) | 2.10E–01 | 3.15E–01 |
| GRAVY | –0.2596 (62) | –0.0979 (90) | **1.10E–04** | **9.87E–04** |
| Theoretical pI | 6.5817 (66) | 7.0990 (78) | **4.90E–02** | 8.52E–02 |
| Charge | –2.4444 (66) | 1.7949 (79) | **3.87E–02** | 8.03E–02 |
| Domain number | 3.3333 (73) | 1.8205 (60) | **2.97E–02** | 6.68E–02 |
| Disorder score | 0.1799 (76) | 0.1160 (54) | **1.72E–03** | **6.65E–03** |
| PEST motif number | 0.6364 (74) | 0.1795 (57) | **2.88E–03** | **9.72E–03** |
| TSPS | 1.3393 (68) | 1.5738 (70) | 4.09E–01 | 4.25E–01 |
| Age | 8.5500 (52) | 9.9138 (63) | 5.05E–02 | 8.52E–02 |
| Evolutionary rate | 5.5031 (54) | 0.3266 (50) | 2.56E–01 | 3.45E–01 |
| Cratio | 38.5784 (62) | 39.3085 (60) | 3.57E–01 | 4.09E–01 |
| Pathway number | 6.1327 (75) | 3.2105 (51) | **6.31E–04** | **2.84E–03** |
| Reaction number | 0.0404 (67) | 0.3077 (75) | **5.02E–03** | **1.35E–02** |
| Degree_PPI | 9.9565 (65) | 8.8889 (63) | 3.65E–01 | 4.09E–01 |
| Betweenness centrality_PPI | 0.0002 (66) | 0.0001 (60) | 1.91E–01 | 3.03E–01 |
| Degree_signal | 40.8778 (69) | 29.3056 (50) | **3.59E–03** | **1.08E–02** |
| Betweenness centrality_signal | 0.0012 (71) | 0.0003 (45) | **1.63E–04** | **1.10E–03** |
| Indegree_TF | 7.4815 (59) | 3.8519 (42) | **6.85E–03** | **1.68E–02** |
| Outdegree_TF | 4.0000 (6) | 19.3333 (7) | 3.78E–01 | 4.09E–01 |

*Note*: ^1^, *P* values smaller than 0.05 are represented in bold type. Adjusted *P* value was computed by Benjamini-Hochberg multiple testing correction method.
