# Supplementary material for: Exploration of Target Spaces in the Human Genome for Protein and Peptide Drugs
Source: Genomics Proteomics Bioinformatics. 2022 Mar 23;20(4):780–94. doi: 10.1016/j.gpb.2021.10.007 (PMC9881050; doi:10.1016/j.gpb.2021.10.007)
Supplement: Supplementary Table S11 [file mmc11.docx]

**Table S11 Qualitative differences between protein and peptide drug targets**

| Property | The fraction of proteins belonging  to a certain protein class (%) | | *P* value  (Fisher’s exact test,  one-sided) ^1^ | Adjusted  *P* value ^1^ |
| --- | --- | --- | --- | --- |
|  | **Protein drug**  **targets** | **Peptide drug**  **targets** |  |  |
| Protein with signal peptide | 84.85 | 51.28 | **7.87E–05** | **7.87E–04** |
| Protein with transmembrane region | 66.67 | 61.54 | 3.53E–01 | 4.23E–01 |
| Signaling molecule | 90.91 | 92.31 | 5.45E–01 | 5.45E–01 |
| Transcription factor | 0.00 | 2.56 | 2.83E–01 | 4.23E–01 |
| Housekeeping gene | 24.24 | 30.77 | 2.81E–01 | 4.23E–01 |
| Self-interacting protein | 22.22 | 17.95 | 3.81E–01 | 4.23E–01 |
| Enzyme | 15.15 | 38.46 | **3.67E–03** | **1.22E–02** |
| GPCR | 5.05 | 28.21 | **4.06E–04** | **2.03E–03** |
| Ion channel | 0.00 | 0.00 | – | – |
| NHR | 0.00 | 2.56 | 2.83E–01 | 4.23E–01 |
| Kinase | 3.03 | 0.00 | 3.66E–01 | 4.23E–01 |
| Transporter | 0.00 | 0.00 | – | – |

*Note*: ^1^, *P* values smaller than 0.05 are represented in bold type. Adjusted *P* value was computed by Benjamini-Hochberg multiple testing correction method.
