# Supplementary material for: Exploration of Target Spaces in the Human Genome for Protein and Peptide Drugs
Source: Genomics Proteomics Bioinformatics. 2022 Mar 23;20(4):780–94. doi: 10.1016/j.gpb.2021.10.007 (PMC9881050; doi:10.1016/j.gpb.2021.10.007)
Supplement: Supplementary Table S12 [file mmc12.docx]

**Table S12 Summary of the features of protein and peptide drug targets**

| Target  type | The number of properties studied in this work (including quantitative and qualitative properties) | The number of features with statistical significance (after multiple testing correction) | The number of features integrated into the final prediction model (the feature list) |
| --- | --- | --- | --- |
| Protein  drug target | 39 | 24 (Tables 1 and 2) | 6 (Betweenness centrality_signal, Transmembrane region, Signal peptide, Housekeeping gene, Indegree_TF, Transporter) |
| Peptide  drug target | 39 | 20 (Tables S6 and S7) | 6 (Signaling molecule, Housekeeping gene, Non-polar, Indegree_TF, GPCR, Signal peptide) |
