# Supplementary material for: Exploration of Target Spaces in the Human Genome for Protein and Peptide Drugs
Source: Genomics Proteomics Bioinformatics. 2022 Mar 23;20(4):780–94. doi: 10.1016/j.gpb.2021.10.007 (PMC9881050; doi:10.1016/j.gpb.2021.10.007)
Supplement: Supplementary Table S14 [file mmc14.docx]

**Table S14 Feature ordering for the target prediction of protein drugs**

| Order | Feature ^1^ |
| --- | --- |
| 1 | Betweenness centrality_signal |
| 2 | Transmembrane region |
| 3 | Signal peptide |
| 4 | Housekeeping gene |
| 5 | Indegree_TF |
| 6 | Transporter |
| 7 | Pathway number |
| 8 | Basic |
| 9 | Signaling molecule |
| 10 | Betweenness centrality_PPI |
| 11 | Domain number |
| 12 | Degree_signal |
| 13 | Age |
| 14 | Charged |
| 15 | TSPS |
| 16 | Self-interacting protein |
| 17 | Small |
| 18 | Degree_PPI |
| 19 | GRAVY |
| 20 | pI |
| 21 | Reaction number |
| 22 | Aromatic |
| 23 | Charge |
| 24 | Tiny |

*Note*: ^1^, These features were ranked using mRMR method based on the gold standard dataset. The GSN set was repeatedly constructed 100 times, and thus, the mRMR feature ranking was implemented based on the mean MI of the 100 times (see Method in the main document). mRMR, minimum redundancy maximum relevance; GSN, gold standard negative; MI, mutual information.
