# Supplementary material for: Exploration of Target Spaces in the Human Genome for Protein and Peptide Drugs
Source: Genomics Proteomics Bioinformatics. 2022 Mar 23;20(4):780–94. doi: 10.1016/j.gpb.2021.10.007 (PMC9881050; doi:10.1016/j.gpb.2021.10.007)
Supplement: Supplementary Table S15 [file mmc15.docx]

**Table S15 Feature ordering for the target prediction of peptide drugs**

| Order | Feature ^1^ |
| --- | --- |
| 1 | Signaling molecule |
| 2 | Housekeeping gene |
| 3 | Non-polar |
| 4 | Indegree_TF |
| 5 | GPCR |
| 6 | Signal peptide |
| 7 | Pathway number |
| 8 | Transmembrane region |
| 9 | Domain number |
| 10 | PEST motif number |
| 11 | Basic |
| 12 | Aromatic |
| 13 | Enzyme |
| 14 | Disorder score |
| 15 | TSPS |
| 16 | Small |
| 17 | Polar |
| 18 | GRAVY |
| 19 | Aliphatic |
| 20 | Charged |

*Note*: ^1^, These features were ranked using mRMR method based on the gold standard dataset. The GSN set was repeatedly constructed 100 times, and thus, the mRMR feature ranking was implemented based on the mean MI of the 100 times (see Method in the main document).
