# Supplementary material for: Exploration of Target Spaces in the Human Genome for Protein and Peptide Drugs
Source: Genomics Proteomics Bioinformatics. 2022 Mar 23;20(4):780–94. doi: 10.1016/j.gpb.2021.10.007 (PMC9881050; doi:10.1016/j.gpb.2021.10.007)
Supplement: Supplementary Table S16 [file mmc16.docx]

**Table S16 ROC AUCs for single-feature target prediction models for protein drugs based on 10-fold cross-validation**

| Single-feature prediction model ^1^ | AUC (mean ± SD) ^1^ |
| --- | --- |
| Betweenness centrality_signal | 0.8478 ± 0.0197 |
| Degree_signal | 0.8460 ± 0.0198 |
| Indegree_TF | 0.8213 ± 0.0173 |
| Pathway number | 0.8170 ± 0.0194 |
| Signal peptide | 0.7968 ± 0.0208 |
| Signaling molecule | 0.7748 ± 0.0241 |
| TSPS | 0.6972 ± 0.0300 |
| Betweenness centrality_PPI | 0.6841 ± 0.0361 |
| Degree_PPI | 0.6815 ± 0.0339 |
| Age | 0.6661 ± 0.0348 |
| Transmembrane region | 0.6604 ± 0.0231 |
| Domain number | 0.6579 ± 0.0245 |
| Basic | 0.6466 ± 0.0290 |
| Charged | 0.6354 ± 0.0338 |
| GRAVY | 0.6245 ± 0.0318 |
| Housekeeping gene | 0.5791 ± 0.0257 |
| pI | 0.5556 ± 0.0376 |
| Charge | 0.5552 ± 0.0330 |
| Small | 0.5530 ± 0.0339 |
| Aromatic | 0.5382 ± 0.0373 |
| Self-interacting protein | 0.5352 ± 0.0184 |
| Tiny | 0.5331 ± 0.0321 |
| Transporter | 0.5089 ± 0.0106 |
| Reaction number | 0.5055 ± 0.0133 |

*Note*: ^1^, The GSN set was repeatedly constructed 100 times, and thus, the presented AUCs are the mean ± SD of the results of the 100 times. The single-feature prediction models are ranked based on the order of decreasing mean value. SD, standard deviation; ROC, receiver operating characteristic; AUC, area under the curve.
