# Supplementary material for: Exploration of Target Spaces in the Human Genome for Protein and Peptide Drugs
Source: Genomics Proteomics Bioinformatics. 2022 Mar 23;20(4):780–94. doi: 10.1016/j.gpb.2021.10.007 (PMC9881050; doi:10.1016/j.gpb.2021.10.007)
Supplement: Supplementary Table S18 [file mmc18.docx]

**Table S18 ROC AUCs of “Model_6_protein” and “Model_6_peptide” against the three independent test sets, in which proteins homologous with those in the gold standard dataset were excluded**

| Assessment method ^1^ | ROC AUC (mean ± SD) ^2^ | |
| --- | --- | --- |
|  | **Model_6_protein** | **Model_6_peptide** |
| Independent test set1 | 0.9534 ± 0.0147 | 0.9176 ± 0.0231 |
| Independent test set2 | 0.9314 ± 0.0082 | 0.9331 ± 0.0154 |
| Independent test set3 | 0.9691 ± 0.0139 | 0.9102 ± 0.0301 |

*Note*: ^1^, The three independent test sets were, respectively, obtained by excluding proteins homologous with those in the gold standard dataset from the three original independent test sets (as described in Method section of the main document). Here we used the same homolog identification method as Ref. 34 (sequence identity > 40%). For the first independent test set, the original independent test positive set was composed of the newly added therapeutic target set of the approved protein/peptide drugs from the latest version of DrugBank (released on July 03, 2018). For the second independent test set, the original independent test positive set was composed of targets of clinical trial protein/peptide drugs [29]. As for the third original independent test set, we divided the GSP set into two parts. One part was composed of targets of protein/peptide drugs approved before 2010, which was used as the positive training set; the other was composed of targets of protein/peptide drugs approved in or after 2010, which was used as the independent positive test set. Please see more details in Method of the main document. ^2^, The GSN set and the independent test negative set were repeatedly constructed 10 times, respectively, and thus the AUC is the mean ± SD of the results of 100 (= 10 × 10) times.
