# Supplementary material for: Exploration of Target Spaces in the Human Genome for Protein and Peptide Drugs
Source: Genomics Proteomics Bioinformatics. 2022 Mar 23;20(4):780–94. doi: 10.1016/j.gpb.2021.10.007 (PMC9881050; doi:10.1016/j.gpb.2021.10.007)
Supplement: Supplementary Table S19 [file mmc19.docx]

**Table S19 ROC AUCs of “Model_6_protein” and “Model_6_peptide” against the three independent test sets, for each of which the independent test negative set was homologous with the positive test set**

| Assessment method ^1^ | ROC AUC (mean ± SD) ^2^ | |
| --- | --- | --- |
|  | **Model_6_protein** | **Model_6_peptide** |
| Independent test set1 | 0.8541 ± 0.0291 | 0.8505 ± 0.0276 |
| Independent test set2 | 0.8189 ± 0.0149 | 0.8192 ± 0.0318 |
| Independent test set3 | 0.8720 ± 0.0291 | 0.7762 ± 0.0220 |

*Note*: ^1^, For each of the independent test sets, every protein in the independent test negative set was homologous with at least one protein in the independent test positive set. Here we used the same homolog identification method as Ref. 34 (sequence identity > 40%). The first independent test positive set was composed of the newly added therapeutic target set of the approved protein/peptide drugs from the latest version of DrugBank (released on July 3, 2018). The second independent test positive set was composed of targets of clinical trial protein/peptide drugs [29]. As for the third independent test set, we divided the GSP set into two parts. One part was composed of targets of protein/peptide drugs approved before 2010, which was used as the positive training set; the other was composed of targets of protein/peptide drugs approved in or after 2010, which was used as the independent positive test set. Please see more details in Method of the main document. ^2^, The GSN set and the independent test negative set were repeatedly constructed 10 times, respectively, and thus the presented ROC AUCs are mean ± SD of results of 100 (= 10 × 10) times.
