# Supplementary material for: Exploration of Target Spaces in the Human Genome for Protein and Peptide Drugs
Source: Genomics Proteomics Bioinformatics. 2022 Mar 23;20(4):780–94. doi: 10.1016/j.gpb.2021.10.007 (PMC9881050; doi:10.1016/j.gpb.2021.10.007)
Supplement: Supplementary Table S20 [file mmc20.docx]

**Table S20 Examples of POPPIT target prediction results**

| Protein name | Gene name  (SwissProt_AC) | Target prediction score (Rank) for a certain type of drug ^1^ | Corresponding  drug (type) | The FDA  approval time | Drug indication |
| --- | --- | --- | --- | --- | --- |
| Interleukin-5 receptor subunit alpha | *IL5RA*  (Q01344) | 7049 (rank = 3) for protein drug | Benralizumab (antibody) | Nov. 2017 | For the add-on maintenance treatment of patients with severe asthma aged 12 years and older ^b^ |
| B-cell receptor CD22 | *CD22*  (P20273) | 2189 (rank = 29) for protein drug | Inotuzumab ozogamicin (antibody-drug conjugate) | Aug. 2017 | For the treatment of adults with relapsed or refractory B-cell precursor acute lymphoblastic leukemia ^c^ |
| Interleukin-4 receptor subunit alpha | *IL4R*  (P24394) | 174 (rank = 361) for protein drug | Dupilumab  (antibody) | Mar. 2017 | For the treatment of adult patients with moderate-to-severe atopic dermatitis whose disease is not adequately controlled with topical prescription therapies or when those therapies are not advisable ^d^ |
| Leptin receptor | *LEPR*  (P48357) | 130 (rank = 364) for protein drug | Metreleptin  (protein, a recombinant analog of the human hormone leptin) | Feb. 2014 | For the treatment of the complications of leptin deficiency in patients with congenital or acquired generalized lipodystrophy ^e^ |
| Melanocortin receptor 4 | *MC4R*  (P32245) | 91 (rank = 278) for peptide drug | Bremelanotide  (peptide) | Jun. 2019 | For the treatment of hypoactive sexual desire disorder in premenopausal women ^f^ |

*Note*: This table presented the examples with high POPPIT prediction scores that actually have had corresponding drugs recently approved by FDA. ^1^, The target prediction score is the combined_LR given by the target prediction model of a certain type of drug. “Rank” was obtained based on the whole genome ranked according to the decreasing prediction scores for a certain type of drug. ^b^ <https://www.accessdata.fda.gov/drugsatfda_docs/label/2017/761070s000lbl.pdf> ^c^ <https://www.accessdata.fda.gov/drugsatfda_docs/label/2017/761040s000lbl.pdf> ^d^ <https://www.accessdata.fda.gov/drugsatfda_docs/label/2017/761055lbl.pdf> ^e^ <https://www.accessdata.fda.gov/drugsatfda_docs/label/2014/125390s000lbl.pdf> ^f^ <https://www.accessdata.fda.gov/drugsatfda_docs/appletter/2019/210557Orig1s000ltr.pdf>. AC, accession number; FDA, the US Food and Drug Administration; LR, likelihood ratio.
