# Supplementary material for: Exploration of Target Spaces in the Human Genome for Protein and Peptide Drugs
Source: Genomics Proteomics Bioinformatics. 2022 Mar 23;20(4):780–94. doi: 10.1016/j.gpb.2021.10.007 (PMC9881050; doi:10.1016/j.gpb.2021.10.007)
Supplement: Supplementary Table S21 [file mmc21.docx]

**Table S21 ROC AUCs of “Model_6_protein” constructed by different machine learning methods**

| Machine learning method ^1^ | ROC AUC (mean ± SD) ^2^ | | | |
| --- | --- | --- | --- | --- |
|  | **10-fold cross validation** | **Independent test set1 ^3^** | **Independent test set2 ^3^** | **Independent test set3 ^3^** |
| NB | 0.9560 ± 0.0098 | 0.9695 ± 0.0101 | 0.9433 ± 0.0080 | 0.9703 ± 0.0142 |
| LR | 0.9412 ± 0.0120 | 0.9445 ± 0.0144 | 0.9216 ± 0.0116 | 0.9582 ± 0.0239 |
| SVM | 0.9190 ± 0.0225 | 0.9381 ± 0.0198 | 0.9221 ± 0.0177 | 0.9575 ± 0.0226 |
| KNN | 0.9227 ± 0.0132 | 0.8902 ± 0.0395 | 0.8995 ± 0.0185 | 0.9424 ± 0.0401 |
| RF | 0.9539 ± 0.0102 | 0.9529 ± 0.0107 | 0.9427 ± 0.0104 | 0.9636 ± 0.0156 |
| DT | 0.8598 ± 0.0296 | 0.8540 ± 0.0335 | 0.8402 ± 0.0218 | 0.8556 ± 0.0418 |
| NN | 0.9303 ± 0.0172 | 0.9216 ± 0.0216 | 0.9186 ± 0.0173 | 0.9344 ± 0.0306 |

*Note*: ^1^, NB, naïve Bayes. SVM, support vector machine. NN, neural network. DT, decision tree. RF, random forest. LR, logistic regression. KNN, K-nearest neighbor. See the details in File S1. ^2^, For the 10-fold cross-validation, the GSN set was repeatedly constructed 50 times, and thus the ROC AUCs are mean ± SD of results of 50 times. For the independent tests, the GSN set and the independent test negative set were repeatedly constructed 10 times, respectively, and thus the presented ROC AUCs are mean ± SD of results of 100 (= 10 × 10) times. ^3^, The first independent test positive set was composed of the newly added therapeutic target set of the approved protein drugs from the latest version of DrugBank (released on July 3, 2018). The second independent test positive set was composed of targets of clinical trial protein drugs [29]. As for the third independent test set, we divided the GSP set into two parts. One part was composed of targets of protein drugs approved before 2010, which was used as the positive training set; the other was composed of targets of protein drugs approved in or after 2010, which was used as the independent positive test set. Please see more details in Method of the main document.
