# Supplementary material for: Exploration of Target Spaces in the Human Genome for Protein and Peptide Drugs
Source: Genomics Proteomics Bioinformatics. 2022 Mar 23;20(4):780–94. doi: 10.1016/j.gpb.2021.10.007 (PMC9881050; doi:10.1016/j.gpb.2021.10.007)
Supplement: Supplementary File S1 — Supplementary methods [file mmc22.docx]

**File S1 Supplementary methods**

**The integrated human protein–protein interaction (PPI) network**

The human PPI network was obtained by integrating experimental PPIs of the “direct interaction” type from multiple databases, including the Database of Interacting Proteins (DIP) (version: April 30, 2016) [1], Molecular INTeraction database (MINT) (downloaded on July 17, 2016) [2], IntAct (version: July 6, 2016) [3], and Biological General Repository for Interaction Datasets (BioGRID) (version: 3.4.138) [4]. The network contained 69,357 PPIs between 12,558 proteins.

**Mutual information**

The mutual information (MI), denoted by *I*, between two discrete random variables *X* and *Y* was computed as:

$$I(X,Y)=\sum_{y\in Y} \sum_{x\in X} p(x,y)\log\left(\frac{p(x,y)}{p(x)p(y)}\right)$$

where *p*(*x*, *y*) is the joint probabilistic distribution, and *p*(*x*) and *p*(*y*) are the marginal probability distribution functions. MI measures the mutual dependence between two random variables, that is, it quantifies the amount of information obtained about one random variable through the other random variable [5].

**Performance assessment of the prediction model**

We used the receiver operating characteristic (ROC) curve to measure the performance of the prediction model based on 10-fold cross-validation and the independent test set.

The ROC curve shows the sensitivity and specificity of the prediction model against different score cutoffs, where the X-axis is 1-specificity and the Y-axis is sensitivity. Sensitivity (*TP*/*T*) and specificity (1-*FP*/*F*) are the correctly classified fractions in the positive (*T*) and negative test sets (*F*), respectively, and true positive (*TP*) and false positive (*FP*) are the predicted positives in *T* and *F*, respectively. A good prediction model has an ROC curve climbing rapidly toward the top left corner of the graph, and its effectiveness can be measured by the area under the curve (AUC). A perfect classifier has an AUC of 1.0, while “AUC = 0.5” means that the prediction model is noninformative. A larger AUC indicates stronger prediction power [5–7].

In the 10-fold cross-validation, the gold standard dataset was averagely and randomly divided into 10 subsets. Nine sets were used as the training set to compute the likelihood ratios (LRs) of the features, and the remaining set was used as the test set to count the number of *TP*s and *FP*s at different values of LR*_cutoff_*. If the LR of a protein given by the prediction model exceeded the LR*_cutoff_*, it was viewed as a predicted positive. This process was repeated 10 times, and the numbers of TPs and FPs against varying LR*_cutoff_* values of the 10 test sets were summed to ultimately draw the ROC curve [5–7].

For the assessment by the independent test set, the whole gold standard set was used as the training set, and the independent test set was used for plotting the ROC curve.

**Data and methods related to Figure 2D**

To analyze and compare the drug–disease relationship for different types of drugs, we used high-confidence drug–disease association data provided by Guney et al. [8], which integrated the data from the MEDication Indication high precision subset (MEDI-HPS) (http://knowledgemap.mc.vanderbilt.edu/research/content/MEDI) [9], the Kyoto Encyclopedia of Genes and Genomes (KEGG) (https://www.kegg.jp/kegg/) [10], and the National Drug File - Resource Terminology (NDF-RT) (https://www.nlm.nih.gov/research/umls/sourcereleasedocs/current/NDFRT/), and were strictly controlled for quality. The approved drug–therapeutic target interaction data were obtained from DrugBank (downloaded on July 26, 2015) (see Method in the main document) [11]. For known gene–disease associations, we used Menche et al.’s scheme [12] to combine data from UniProtKB/Swiss-Prot (gene–OMIM disease associations, downloaded on March 23, 2016) [13] and Phenotype–Genotype Integrator (PheGenI) (downloaded on October 21, 2016) [14].

**Data and methods related to “****Disease relevance analysis” of POPPIT**

The relevance between a protein and a disease was measured by their distance in the PPI network [15], which is defined as the minimum shortest path length between the protein and the disease’s known related genes. A distance of zero means that the protein happens to be the disease’s known related gene.

Here the integrated PPI network (as described above) was used. Known gene–disease associations were from three databases including the Online Mendelian Inheritance in Man (OMIM) [16], PheGenI (downloaded on October 21, 2016) [14], and the Comparative Toxicogenomics Database (CTD) (version: July 6, 2017) [17]. For OMIM, we directly extracted gene–OMIM disease associations from Swiss-Prot (downloaded on March 23, 2016) [13]. For PheGenI, we used its genome-wide association study (GWAS) data to obtain gene–disease associations, and only genes with a genome-wide significance *P* value < 5 × 10^–8^ were included. Considering the tree structure of Medical Subject Headings (MeSH) vocabulary (https://www.ncbi.nlm.nih.gov/mesh) which was used to denote diseases in PheGenI, we parsed PheGenI gene–disease association data based on the following rule: if a gene was known to be associated with a MeSH term in PheGenI, it was also thought to be related to all father terms of this MeSH term. Finally, for CTD, we only used its gene–disease associations with direct evidence. And because CTD used MErged DIsease voCabulary (MEDIC) vocabulary [18] also with a tree structure to denote diseases, which is a modified subset of MeSH “Diseases” branch combined with OMIM diseases, we similarly used the rule mentioned above to parse CTD gene–disease association data. In POPPIT, we have not merged the known gene–disease association data from these three databases. Users can specify the gene–disease association data source to perform the “Disease relevance analysis”.

**The implementation of different machine learning methods**

Different machine learning methods were used to construct the protein drug target prediction model (“Model_6_protein”).

For the logistic regression (LR), the data preprocessing included coding for categorical features (for example the feature “transmembrane region” can be represented as 1 and 0) and feature n[ormalization](javascript:;) using Z-score. We used the “*glm*” function in R [19] to implement the LR model construction.

For the support vector machine (SVM), the data preprocessing included coding for categorical features (for example the feature “transmembrane region” can be represented as (1, 0) and (0, 1)) and feature scaling using linear scaling to range [0,1], which were implemented using related programs provided by LIBSVM (<https://www.csie.ntu.edu.tw/~cjlin/libsvm/>) [20]. We used the “radial basis function” (RBF) kernel function recommended by LIBSVM. The optimal parameter *C* and *γ* were trained using ROC AUC as the evaluation criterion, by “*grid.py*” program provided by LIBSVM.

For the K-nearest neighbor (KNN), the data preprocessing included coding for categorical features (for example the feature “transmembrane region” can be represented as 1 and 0) and feature n[ormalization](javascript:;) using Z-score. The optimal parameter *K* was trained using ROC AUC as the evaluation criterion, ranging from [3,$\sqrt{N}$] (step length = 2), where *N* is the number of samples in the training set. KNN model was established by R package “*class*” [21].

For the decision tree (DT) and the random forest (RF), we used R package “*mlr*” [22] to implement it and optimized the parameter “*mtry*” (ranging from 2 to 6) and “*nodesize*” (ranging from 1 to 21, step length = 2). For the DT, “*ntree*” was set to 1, and for the RF, was set to 500. The other parameters were set to default values.

For the neural network (NN), we used Z-score for the feature n[ormalization](javascript:;). We used R package “*neuralnet*” to implement the NN model construction (https://github.com/bips-hb/neuralnet). By optimization, “*hidden*” (*i.e.*, the number of neurons in the hidden layer) was set to 5, “*algorithm*” was set to “slr” (*i.e.*, resilient backpropagation of the modified globally convergent version [23]). The other parameters were set to default values.

**References**

[1] Xenarios I, Salwínski L, Duan XJ, Higney P, Kim SM, Eisenberg D. DIP, the Database of Interacting Proteins: a research tool for studying cellular networks of protein interactions. Nucleic Acids Res 2002;30:303–5.

[2] Licata L, Briganti L, Peluso D, Perfetto L, Iannuccelli M, Galeota E, et al. MINT, the molecular interaction database: 2012 update. Nucleic Acids Res 2012;40:D857–61.

[3] Kerrien S, Aranda B, Breuza L, Bridge A, Broackes-Carter F, Chen C, et al. The IntAct molecular interaction database in 2012. Nucleic Acids Res 2012;40:D841–6.

[4] Chatr-Aryamontri A, Oughtred R, Boucher L, Rust J, Chang C, Kolas NK, et al. The BioGRID interaction database: 2017 update. Nucleic Acids Res 2017;45:D369–79.

[5] Liu Z, Guo F, Zhang J, Wang J, Lu L, Li D, et al. Proteome-wide prediction of self-interacting proteins based on multiple properties. Mol Cell Proteomics 2013;12:1689–700.

[6] Li D, Liu W, Liu Z, Wang J, Liu Q, Zhu Y, et al. PRINCESS, a protein interaction confidence evaluation system with multiple data sources. Mol Cell Proteomics 2008;7:1043–52.

[7] Liu Z, Guo F, Gu J, Wang Y, Li Y, Wang D, et al. Similarity-based prediction for Anatomical Therapeutic Chemical classification of drugs by integrating multiple data sources. Bioinformatics 2015;31:1788–95.

[8] Guney E, Menche J, Vidal M, Barábasi AL. Network-based in silico drug efficacy screening. Nat Commun 2016;7:10331.

[9] Wei WQ, Cronin RM, Xu H, Lasko TA, Bastarache L, Denny JC. Development and evaluation of an ensemble resource linking medications to their indications. J Am Med Inform Assoc 2013;20:954–61.

[10] Kanehisa M, Furumichi M, Tanabe M, Sato Y, Morishima K. KEGG: new perspectives on genomes, pathways, diseases and drugs. Nucleic Acids Res 2017;45:D353–61.

[11] Wishart DS, Feunang YD, Guo AC, Lo EJ, Marcu A, Grant JR, et al. DrugBank 5.0: a major update to the DrugBank database for 2018. Nucleic Acids Res 2018;46:D1074–82.

[12] Menche J, Sharma A, Kitsak M, Ghiassian SD, Vidal M, Loscalzo J, et al. Disease networks. uncovering disease-disease relationships through the incomplete interactome. Science 2015;347:1257601.

[13] Pundir S, Martin MJ, O'Donovan C. UniProt Protein Knowledgebase. Methods Mol Biol 2017;1558:41–55.

[14] Ramos EM, Hoffman D, Junkins HA, Maglott D, Phan L, Sherry ST, et al. Phenotype-Genotype Integrator (PheGenI): synthesizing genome-wide association study (GWAS) data with existing genomic resources. Eur J Hum Genet 2014;22:144–7.

[15] Yildirim MA, Goh KI, Cusick ME, Barabási AL, Vidal M. Drug-target network. Nat Biotechnol 2007;25:1119–26.

[16] Amberger JS, Bocchini CA, Schiettecatte F, Scott AF, Hamosh A. OMIM.org: Online Mendelian Inheritance in Man (OMIM), an online catalog of human genes and genetic disorders. Nucleic Acids Res 2015;43:D789–98.

[17] Davis AP, Grondin CJ, Johnson RJ, Sciaky D, King BL, McMorran R, et al. The Comparative Toxicogenomics Database: update 2017. Nucleic Acids Res 2017;45:D972–8.

[18] Davis AP, Wiegers TC, Rosenstein MC, Mattingly CJ. MEDIC: a practical disease vocabulary used at the Comparative Toxicogenomics Database. Database (Oxford) 2012;2012:bar065.

[19] R Core Team. R: a language and environment for statistical computing. Vienna: R Foundation for Statistical Computing; 2018.

[20] Chang CC, Lin CJ. LIBSVM: a library for support vector machines. ACM T Intel Syst Tec 2011;2:1–27.

[21] Venables WN, Ripley BD. Modern applied statistics with S. 4th ed. New York: Springer; 2002.

[22] Bischl B, Lang M, Kotthoff L, Schiffner J, Richter J, Studerus E, et al. mlr: machine learning in R. J Mach Learn Res 2016;17:1–5.

[23] Anastasiadis A, Magoulas CD, Vrahatis MN. New globally convergent training scheme based on the resilient propagation algorithm. Neurocomputing 2005;64:253–70.
